# Identification and characterization of two novel *Acinetobacter* species capable of degrading polyester

**DOI:** 10.1128/spectrum.02491-25

**Published:** 2026-04-09

**Authors:** Mengli Xia, Yuandong Zhao, Yiran Ma, Bo Wu, Guoquan Hu, Yanwei Wang, Mingxiong He

**Affiliations:** 1 Biomass Energy Technology Research Centre, Biogas Institute of Ministry of Agriculture and Rural Affairs, Chengdu, China; 2 Graduate School of Chinese Academy of Agricultural Science, Beijing, China; 3 Key Laboratory of Development and Application of Rural Renewable Energy, Ministry of Agriculture and Rural Affairs of the People’s Republic of China, Chengdu, China; Connecticut Agricultural Experiment Station, New Haven, Connecticut, USA

**Keywords:** *Acinetobacter minutum *sp. nov., *Acinetobacter kanungonis *subsp. *fragariae *subsp. nov., taxonomic description, plastic degradation, multicopper oxidase, alkane oxidase

## Abstract

The ubiquitous presence of *Acinetobacter* species in the environment holds significant biotechnology potential, particularly in the degradation of various pollutants. In this study, we characterize two plastic-degrading bacteria, strains CAAS 2-6 and CAAS 2-13, isolated from landfill leachate and a strawberry farmland, respectively. Strain CAAS 2-6 showed the highest *16S rRNA* sequence similarity (97.7%) with *Acinetobacter gerneri* DSM 14967^T^, while strain CAAS 2-13 was most closely related (99.6%) to *Acinetobacter kanungonis* PS-1^T^. Phylogenetic analysis of *16S rRNA* and *gyrB-rpoB* genes placed both strains on distinct branches. Genomic comparisons revealed the highest digital DNA-DNA hybridization/average nucleotide identity values for CAAS 2-6 with *Acinetobacter indicus* CIP 110367^T^ (22.7%/77.0%) and for strain CAAS 2-13 with *A. kanungonis* PS-1^T^ (66.5%/96.0%). Based on phenotypic and chemotaxonomic data, we propose strain CAAS 2-6^T^ as the novel species *Acinetobacter minutum* sp. nov. (type strain CAAS 2-6^T^ = GDMCC 1.3951^T^ = JCM 36321^T^ = KCTC 8156^T^), and strain CAAS 2-13^T^ as the novel subspecies *A. kanungonis* subsp. *fragariae* subsp. nov. (strain CAAS 2-13^T^ = GDMCC 1.3956^T^ = JCM 36322^T^ = KCTC 8157^T^). Both strains utilized polylactic acid, polybutylene succinate-adipic acid (PBSA), polybutylene succinate (PBS), polybutylene adipate terephthalate (PBAT), and polybutylene terephthalate as sole carbon sources, with CAAS 2-6 exhibiting superior growth on PBSA, PBS, and PBAT. Genomic annotation identified genes encoding plastic-degrading enzymes multicopper oxidase AbMCO and alkane hydroxylase AlkB. Enzymatic depolymerization assays confirmed *in vitro* production of plastic monomers. These findings expand the known metabolic capabilities of *Acinetobacter* and provide promising new candidates for plastic bioremediation.

**IMPORTANCE:**

*Acinetobacter* spp. demonstrate significant potential for the bioremediation of environmental pollutants. This study discovers and characterizes two novel *Acinetobacter* strains, CAAS 2-6^T^ (*A. minutum* sp. nov.) and new subspecies CAAS 2-13^T^ (*A. kanungonis* subsp. *fragariae* subsp. nov.), which exhibit a remarkable capacity to utilize five major commercial polyesters (polylactic acid, polybutylene adipate terephthalate, polybutylene terephthalate, polybutylene succinate, and polybutylene succinate-adipic acid) as sole carbon sources. Genomic and enzymatic analyses identified a multicopper oxidase (AbMCO) and an alkane hydroxylase (AlkB) as key biocatalysts in the depolymerization process. Our work: (i) significantly expands the known diversity of plastic-degrading *Acinetobacter*, (ii) provides novel enzyme candidates and genomic resources for biocatalyst development, and (iii) supplies engineerable microbial chassis for synthetic biology applications in waste bioconversion.

## INTRODUCTION

The genus *Acinetobacter*, introduced by Brisou and Prévot (1), belongs to the family *Moraxellaceae*, order *Moraxellales*, and class *Gammaproteobacteria*. This taxonomically diverse and ubiquitous genus comprises 87 species with validly published names (https://lpsn.dsmz.de/genus/acinetobacter; last accessed 12 October 2025). *Acinetobacter* spp. are gram-negative, aerobic, catalase-positive, and oxidase-negative, with genomic G + C content of 34.9–47.0% (2). They inhabit diverse environments, including soil (3–8), water ecosystems (2, 9–12), plants (13, 14), animals (15–20), and hospital environments (21–23) (apps.szu.cz/anemec/Classification.pdf), reflecting broad metabolic versatility (24).

Several *Acinetobacter* species exhibit significant biotechnological potential through the degradation of diverse pollutants, including plastics, hydrocarbons, pesticides, and plasticizers. *Acinetobacter* spp. metabolizing pesticides (methyl parathion [25], methamidophos [26], fenamidophos [27], and bensulfuron-methyl [28]), and diesel alkanes (e.g., *A. baumannii* degrading 58.1% in 10 days [29]; *A. vivianii* KJ-1 utilizing diesel as sole carbon source [30]; *A. junii* degrading 57.5% in 45 days [31]; *A. venetianus* RAG-1 participates in long-chain alkane degradation via alcohol dehydrogenases (ADHs) [32]; and *A. haemolyticus* JS-1 degrades 70–80% of crude oil at concentrations of 10–20 g/L within 15 days at 30°C [33]). Strains degrading phthalate esters (e.g., *Acinetobacter* sp. SN13 on di(2-ethylhexyl) phthalate [34]; strain LMB-5 on multiple phthalates [35]). Plastic-degrading strains such as *A. gerneri* P7 (polyurethane via 66-kDa esterase [36]), *A. pittii* IRN19 (untreated LDPE [37]), *Acinetobacter* sp. AnTc-1 (polystyrene [38]), and gut-derived *Acinetobacter* sp. BIT-H3 (rubber [39]). Enzymatic drivers like AlkB (alkane degradation [30, 40]) and AbMCO (polyethylene degradation [41]). The novel species *A. suaedae* sp. nov. preferentially degrades phenolic acids (42), further highlighting metabolic versatility. Despite their metabolic diversity, only a limited number of *Acinetobacter* species have been definitively associated with plastic degradation, and the taxonomic relationship between degradation capacity and phylogenetic position remains unclear. This knowledge gap underscores the need for polyphasic characterization of novel plastic-degrading isolates.

Improper plastic disposal threatens ecosystems and human health (43). While microbial degradation offers a promising remediation strategy (44, 45), key resources are underexplored. Landfills store 21–42% of global plastic waste (46), and agricultural soils accumulate plastic film residues (47); both environments likely harbor specialized plastic-degrading microbes. Despite this potential, *Acinetobacter* strains capable of metabolizing multiple plastics remain scarce, and their enzymatic machinery is poorly characterized.

Here, we identify and characterize two novel *Acinetobacter* taxa with multi-plastic degradation capabilities: *Acinetobacter minutum* sp. nov. CAAS 2-6^T^ and *Acinetobacter kanungonis* subsp. *fragariae* subsp. nov. CAAS 2-13^T^. Beyond taxonomic description, we mined their genomes and confirmed the function of two key catabolic genes (*abMCO* and *alkB*) via heterologous expression, demonstrating their direct involvement in polymer breakdown. Our integrated approach provides the first validated genetic basis for plastic biodegradation in this genus, offering new insights and microbial resources for plastic waste remediation.

## MATERIALS AND METHODS

### Reagents and culture media

This study utilized a plastic film made of polylactic acid (PLA) donated by Sichuan Shennong Chinese Medicine Agricultural Research Institute Group Co., Ltd., and six types of plastics were utilized. Among them, PLA was sourced from NatureWorks LLC in the United States. Polybutylene adipate terephthalate (PBAT) and polybutylene succinate-adipic acid (PBSA) were produced by Xinjiang Lanshantunhe Science and Technology Co., Ltd in China. Polyethylene terephthalate (PET) came from DuPont de Nemours, Inc. in the United States. Polybutylene terephthalate (PBT) was manufactured by Chang Chun Plastics and Chemical Industry Co., Ltd. in China, and polybutylene succinate (PBS) was supplied by PTT MCC Biochem Co., Ltd. in Thailand. Other chemical reagents were purchased from Sinopharm Chemical Reagent Co., Ltd. and Shanghai Macklin Biochemical Co., Ltd. in China. KOD One polymerase was purchased from Takara Biotechnology Co., Ltd. in Japan. Mineral salt medium (MSM) consisted of the following (g/L): 7.0 K_2_HPO_4_, 2.0 KH_2_PO_4_, 1.0 NH_4_NO_3_, 0.1 MgSO_4_·7H_2_O, 0.01 FeSO_4_·7H_2_O, 0.001 ZnSO_4_·7H_2_O, 0.0002 MnSO_4_·6H_2_O, and 0.0001 CuSO_4_·7H_2_O (pH 7.5) (48–50). Add 1.0 g/L yeast extract as a growth factor if necessary. Luria-Bertani (LB) medium consisted of the following (g/L): 10.0 Tryptone, 5.0 yeast extract, and 5.0 NaCl. The solid medium was supplemented with 15 g/L agar and sterilized at 121°C for 20 min.

### Strain isolation and molecular identification

This study conducted sampling from various environments in Chengdu, Sichuan Province, China. Two grams of each sample was enriched in five rounds, with each round lasting for 7 days, in 25 mL of MSM medium supplemented with PLA film. The PLA film was sterilized by soaking in 75% ethanol for 10 min and then air-dried under UV light. Enrichment and subculturing were performed by transferring 2 mL of the pelagic microbes and PLA film from the previous culture to fresh 25 mL of MSM. Following the completion of the fifth enrichment round, *Acinetobacter* species were isolated and purified from the culture fluid. The standard dilution plating method was employed to spread the enriched culture onto LB agar plates (51). After incubation at 30°C for 72 h, individual colonies were randomly selected and repeatedly streaked on LB agar plates to obtain pure cultures. The selected purified isolates were cryopreserved in a suspension containing 20% (vol/vol) glycerol at −80°C.

For phylogenetic analysis of the *16S rRNA* gene, PCR amplification and sequencing were carried out following a previously described method (52). Subsequent BLAST analysis was performed for the *16S rRNA* gene sequences via the EzBioCloud web service (53). The *16S rRNA* gene sequences of strains CAAS 2-6 and CAAS 2-13, along with 99 typical strains of the genus *Acinetobacter* available in the NCBI database, were aligned using CLUSTAL X 2.0 software (54). The aligned sequences were used in the program MEGA 11.0 (55) to construct the neighbor-joining (NJ) (56) and maximum-likelihood (ML) (57) phylogenetic trees using the Maximum Composite Likelihood and Tamura-Nei model, with bootstrap values calculated from 1,000 replications.

### Phenotypic and chemotaxonomic characterization

Colony morphology was observed on LB agar after 48 h of incubation at 37°C. Cell motility was evaluated by stab inoculation in semi-solid medium containing 0.5% (wt/vol) agar. The Gram reaction was determined using a commercial Gram staining kit (G1060, Beijing Solaibao Technology, China). Anaerobic growth was assessed in Mitsubishi AnaeroPouch-Bag systems over 7 days incubation at 30°C. Cell morphology was examined by scanning electron microscopy (Hitachi SU8010, Japan) using mid-log phase cells grown in LB broth at 30°C.

Growth characteristics were determined by measuring the optical density at 600 nm (L5S, INESA, China) in LB medium after 48 h under various conditions: temperatures from 4°C to 60°C (4°C, 8°C, 16°C, 20°C, 25°C, 30°C, 37°C, 40°C, 45°C, 50°C, and 60°C), pH levels from 4.0 to 11.0 (in increments of 0.5 pH units, adjusted with biological buffers), and NaCl concentrations from 0% to 10% (wt/vol) (in 1.0% intervals). Growth curves were constructed by inoculating single colonies into LB broth and monitoring the OD_600_ at 0, 0.5, 1, 2, 3, 4, 5, 6, 7, 8, 9, 10, 11, and 12 h. All physiological assays were performed in biological triplicates.

Catalase activity was confirmed by bubble formation in 3% (vol/vol) hydrogen peroxide, and oxidase activity was detected using 1% (wt/vol) tetramethyl-ρ-phenylenediamine. Carbohydrate acid production was analyzed with the API 50CH system (bioMérieux), while other biochemical and enzymatic properties were tested using the API 20E, API 20NE, and API ZYM kits (bioMérieux) according to the manufacturer’s protocols.

For whole-cell fatty acid analysis, strains were cultivated on LB agar at 30°C for 48 h. Fatty acids were extracted and analyzed following the standard protocols of the Microbial Identification System (MIDI) (58) and separated using an Agilent 6890N gas chromatograph. For polar lipid analysis, cells were harvested during the mid-exponential phase from LB broth cultured at 30°C with shaking. Polar lipids were extracted from 200 mg of lyophilized cells using the Bligh-Dyer method (59) and separated by two-dimensional thin-layer chromatography on silica gel GF254 plates (100 × 100 mm, Qingdao Marine Chemical Factory, China) under the chromatographic conditions described by Minnikin et al. (60–62). Definitive lipid identification and validation were conducted by the China Agricultural Culture Collection Center using standardized microbial chemotaxonomic procedures.

### Whole-genome sequencing and analysis

The draft genome was sequenced by Shanghai Pasino Biotechnology Co., Ltd. (China) using the Whole Genome Shotgun strategy. Libraries with varying insert sizes were constructed and sequenced on the Illumina NovaSeq platform via paired-end sequencing. Raw reads were processed as follows: AdapterRemoval (63) was used to trim adapter contaminants, and SOAPec (64) was employed for quality correction. The cleaned reads were then assembled *de novo* using A5-MiSeq version 20,160,825 (65) with SPAdes v3.12.0 (66) (https://github.com/ablab/spades/releases). The resulting assembly was polished with Pilon software v1.18 (67) for base-level corrections. Gene prediction and annotation were performed using GeneMarkS v4.32, Barrnap v0.9, tRNAscan-SE v1.3.1, and CRISPRCasFinder v4.2.20 for predicting and annotating open reading frames, rRNA, tRNA, and CRISPRs (68–71). Protein-coding sequences were functionally annotated by aligning against the NCBI NR, eggNOG (evolutionary genealogy of genes: Non-supervised Orthologous Groups), KEGG (Kyoto Encyclopedia of Genes and Genomes), Swiss-Prot, and GO (Gene Ontology) databases using diamond blastp.

For phylogenetic placement, strains CAAS 2-6 and CAAS 2-13 were initially analyzed using the Type Strain Genome Server (TYGS) tool (https://tygs.dsmz.de, accessed 20 October 2023) (72). Digital DNA-DNA hybridization (dDDH) and average nucleotide identity (ANI) values between these strains and their close relatives were calculated via the EzBioCloud web server (https://www.ezbiocloud.net/tools/ani) (53) and the Genome-to-Genome Distance Calculator v3.0 using the recommended Formula 2 (http://ggdc.dsmz.de) (73). Genome-wide relatedness was further assessed using BLAST-based ANI (ANIb) (74) and OrthoANI (75). Finally, pan-genome analysis and genomic collinearity analysis were performed for six *Acinetobacter* strains: *Acinetobacter* sp. CAAS 2-6, *Acinetobacter* sp. CAAS 2-13, *A. chinensis* WCHAc010005^T^, *A. towneri* GX3^T^, *A. gerneri* 9A01^T^, and *A. kanungonis* PS1^T^, using IPGA v1.09 (https://nmdc.cn/ipga) (76) and TBtools-II v2.225 (77).

### Growth studies in modified MSM supplemented with plastics

Cells were cultured in LB medium at 30°C to mid-log phase (OD_600_ ≈ 0.6), harvested by centrifugation (4,000 × *g*, 3 min, 4°C), washed three times with sterile 0.85% (wt/vol) NaCl, and resuspended in modified MSM (1.0 g/L yeast extract). For growth utilization assays, saline-washed cells (OD_600_ adjusted to 0.8 ± 0.05) were inoculated at 10% (vol/vol) into 9 mL of MSM with 1.0 g/L yeast extract supplemented with 0.2 g/L plastic powder (<100 µm particle size) as the sole carbon source. Cultures were incubated at 30°C with shaking (180 rpm) for 7 days. Biomass accumulation was monitored at OD_600_, followed by high-performance liquid chromatography (HPLC) analysis of culture supernatants for monomer quantification.

The liquid samples were centrifuged at 12,000 × *g* for 10 min and filtered through 0.22 μm membranes into HPLC vials. Analytical protocols were tailored to monomer types by selecting distinct instruments, columns, and programs. For nonpolar or weakly polar compounds, a Shimadzu LC-16 HPLC system equipped with an Agilent HC-C18 column (4.6 × 250 mm, 5 μm particle size) was employed. The mobile phase consisted of 0.1% (vol/vol) formic acid and acetonitrile under gradient elution: 70% formic acid/30% acetonitrile (0–10 min), linear gradient to 100% acetonitrile (10–20 min), and 100% acetonitrile (20–30 min). The injection volume was 20 μL, column temperature maintained at 30°C, and flow rate set to 1.0 mL/min, with effluent monitored at 254 nm using a diode array detector. For polar compounds, an Agilent 1200 HPLC system with an Aminex HPX-87H column (300 × 7.6 mm) was utilized. Isocratic elution was performed over 30 min using 50 mM sulfuric acid as the mobile phase. The injection volume was 20 μL, column temperature held at 35°C, and flow rate adjusted to 0.6 mL/min, with effluent detected by a refractive index detector. Calibration curves were constructed using standard solutions of terephthalic acid (TPA), lactic acid (LA), 1,4-butanediol (BDO), adipic acid (AA), and succinic acid (SA) at concentrations of 0.05, 0.1, 0.25, 0.5, 1.0, 2.5, and 5.0 g/L. Triplicate injections were performed for each concentration, and linear regression analyses yielded correlation coefficients *R*^2^ > 0.999 (Fig. S5).

Furthermore, microbial degradation was assessed using pure PLA film (~0.5 g/L) as the sole carbon source and substrate. The post-degradation films were characterized by weight loss measurement, thermogravimetric (TG), and Fourier-transform infrared (FTIR) spectroscopy. Weight loss was determined using an ultra-microbalance (readability: 0.000001 g) after the films were rinsed with 0.1% (wt/vol) SDS solution and thoroughly dried. TG was performed on a NETZSCH TG 209 F1 Libra thermogravimetric analyzer (NETZSCH, Germany). Samples weighing 2.5–8 mg were placed in open alumina crucibles and heated from 30°C to 600°C at a rate of 10°C·min^−1^ under a high-purity nitrogen (99.999%) atmosphere with a flow rate of 20 mL·min^−1^. The temperature corresponding to the maximum rate of weight loss (*Td*) was derived from the first derivative thermogravimetric (DTG) curve. FTIR spectra of the films before and after degradation were acquired using a Bruker INVENIO-S spectrometer (Bruker, Germany) equipped with an ATR accessory. Spectra were recorded in the range of 4000–400 cm^−1^ at a resolution of 2 cm^−1^ by accumulating 32 scans per measurement.

### Gene cloning, protein purification, and enzymatic degradation of plastic powder

Gene sequences encoding putative multicopper oxidase AbMCO (EC 1.3.3.5) and alkane hydroxylase AlkB (EC 1.14.15.3) homologs were selected for molecular cloning and heterologous expression. Target genes were PCR-amplified using gene-specific primers (Table S4; synthesized by Sangon Biotech, Shanghai) and subsequently cloned into the pET28a(+) expression vector via Gibson assembly. The recombinant plasmids were transformed into *Escherichia coli* BL21(DE3) cells for polyhistidine-tagged protein purification as follows:

1. Primary seed cultures were grown overnight at 37°C with 200 rpm shaking in 5 mL LB broth supplemented with kanamycin (0.1 g/L). Secondary cultures were inoculated at 1:50 dilution in 50 mL kanamycin-supplemented LB broth (0.1 g/L) and incubated at 37°C (200 rpm) until OD_600_ reached 0.6–0.8. Protein expression was induced with 1 mM IPTG at 25°C for 10 h (200 rpm). Cells were harvested by centrifugation at 4,000 × *g* for 10 min (4°C; TGL-21M centrifuge, Luxiangyi).
2. Cell pellets were resuspended in lysis buffer and disrupted by ultrasonication (250 W; 5 s pulse-on/5 s pulse-off cycles for 15 min total; SCIENTZ-IID, Xinzhi). Lysates were clarified by centrifugation at 20,000 × *g* for 20 min (4°C; TGear HC16R, Tiangen). Supernatants were subjected to affinity purification using Ni-NTA gravity columns (HyPur T 6FF; Sangon Biotech, C600791-0010) according to the manufacturer’s protocols.
3. Protein expression and purity were verified by SDS-PAGE. Concentrations were determined via modified Bradford assay (Sangon Biotech, C503041-1000) using bovine serum albumin (BSA) standards.
4. A 200 μL aliquot of the nickel column-purified protein solution was mixed with 800 μL of either 50 mM sodium acetate buffer (pH 6.0, 3.88 g/L CH_3_COONa and 0.155 mL/L CH_3_COOH) or 50 mM Tris-HCl buffer (pH 7.5, 6.06 g/L Tris and 3.33 mL/L HCl) supplemented with 1 mM NADH. The reaction mixtures were incubated at 37°C with agitation (180 rpm) for 5 days. After incubation, the supernatants of the enzymatic hydrolysates were collected, filter-sterilized, and initially analyzed by HPLC for the quantification of suspected plastic monomers, such as TPA, LA, BDO, AA, and SA.

### Untargeted metabolomic profiling by high-resolution liquid chromatography-mass spectrometry (LC-MS)

Following the initial HPLC screening, a comprehensive, untargeted analysis was conducted to identify metabolic intermediates derived from the enzymatic depolymerization. The sample preparation involved liquid-liquid extraction: 2 mL of the filtered hydrolysate was combined with 400 µL of methanol. The extracted solution was dried, and the residue was reconstituted in 150 µL of 80% methanol/water containing 4 ppm 2-chlorophenylalanine as an internal standard for quality control. The reconstituted solution was centrifuged, and the supernatant was filtered prior to LC-MS analysis.

The analysis was performed on a high-resolution LC-MS platform consisting of a Waters ACQUITY UPLC system coupled to a Thermo Q Exactive mass spectrometer. Separation was achieved on an ACQUITY UPLC HSS T3 column (2.1 × 150 mm, 1.8 µm) at 40°C. An injection volume of 2 µL was eluted at 0.25 mL/min. For positive ion mode, the mobile phases consisted of 0.1% formic acid in water (A1) and 0.1% formic acid in acetonitrile (B1). The gradient program was as follows: 2% B1 (0–1 min), 2–50% B1 (1–9 min), 50–98% B1 (9–12 min), 98% B1 (12–13.5 min), 98–2% B1 (13.5–14 min), and 2% B1 (14–20 min). For negative ion mode, the mobile phases were 5 mM ammonium formate in water (A2) and acetonitrile (B2). The gradient program was: 2% B2 (0–1 min), 2–50% B2 (1–9 min), 50–98% B2 (9–12 min), 98% B2 (12–13.5 min), 98–2% B2 (13.5–14 min), and 2% B2 (14–17 min).

To achieve optimal detection for a broad range of metabolites, data were acquired in both positive and negative electrospray ionization modes with spray voltages of 3.50 and −2.50 kV, respectively. The sheath gas and auxiliary gas flow rates were set at 30 and 10 arbitrary units, respectively. The capillary temperature was 325°C. The instrument operated in full MS/dd-MS^2^ mode: a full scan at 70,000 resolution (*m/z* = 100–1,000) was followed by data-dependent MS/MS scans at 17,500 resolution on the top 10 ions using HCD fragmentation (30 eV). Dynamic exclusion was implemented to enhance the coverage of lower-abundance ions.

### Modeling and molecular docking

Protein structure prediction was performed using AlphaFold 3 (78) via Google Colaboratory, and the optimal model was validated using Ramachandran plot analysis. Substrate molecules were sketched in ChemDraw 3D (v20.0) and exported as PDB files. Molecular docking was conducted in AutoDock Vina (79, 80) following hydrogen addition to both the predicted enzyme structure and substrate. Three-dimensional (3D) structural visualization and docking analyses were performed using PyMOL (v2.6).

Molecular docking studies were performed using oligomers composed of six monomer candidates—TPA (T), BDO (B), ethylene glycol (E), AA (A), SA (S), and LA (L)—as substrates. The selected oligomers (ABT, BAB, BABTB, BSB, BT, BTB, BTBT, LLLL, MHET, MHET_4_, TBT, and TBTBT) were docked against the predicted 3D structures of AlkB enzyme from strain CAAS 2-13 and AbMCO1 enzyme from strain CAAS 2-6. To determine the conserved domain characteristics, the protein structures of the same enzyme from different species within the same genus were also compared.

## RESULTS AND DISCUSSION

### Isolation, morphology, and chemotaxonomic features

**Fig 1:**
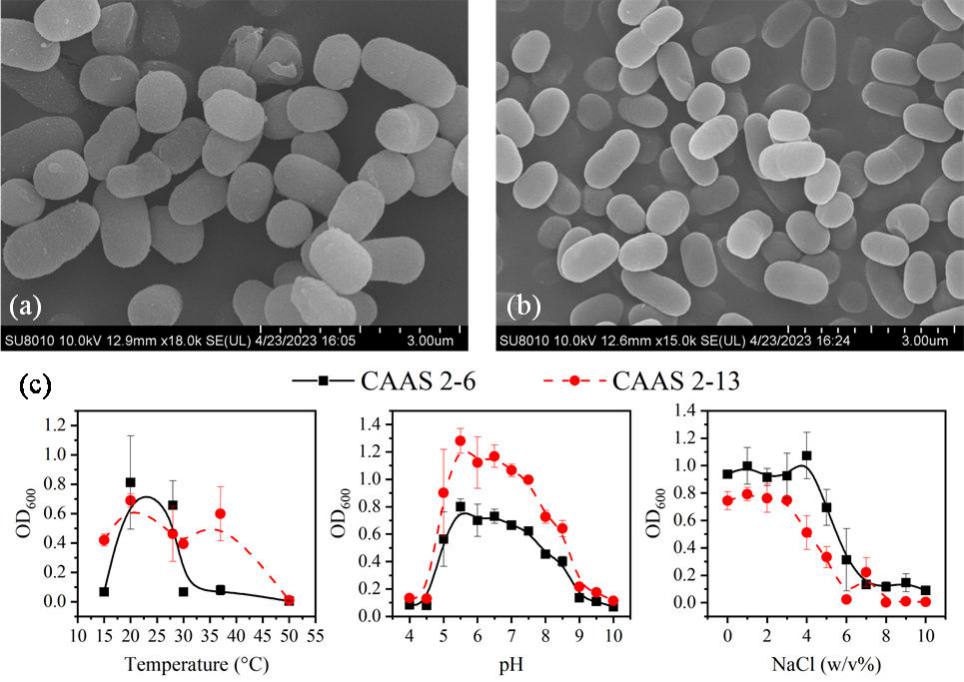
Morphological and physiological characterization of strains CAAS 2-6 and CAAS 2-13. (**a and b**) Scanning electron micrographs of CAAS 2-6 and CAAS 2-13, respectively, illustrating their rod-shaped morphology with dimensions of 0.6–0.7 µm × 0.7–1.3 µm and 0.5–0.6 µm × 0.7–1.2 µm. (**c**) Optimal growth conditions for both strains, determined by assessing growth at various temperatures, pH levels, and NaCl concentrations. Scale bars, 3 μm.

The strain CAAS 2-6 was isolated from leachate of Chang'an Landfill (coordinates: 30°65′35.38″ N, 104°37′81.79″ E) in the Longquan Mountain Range, Chengdu, China, while strain CAAS 2-13 originated from a strawberry cultivation field (coordinates: 30°40′92.42″ N, 104°15′15.72″ E) on the Chengdu Plain. The cells were Gram-stain-negative, aerobic, and short rods with dimensions of 0.6–0.7 µm × 0.7–1.3 µm (CAAS 2-6) and 0.5–0.6 µm × 0.7–1.2 µm (CAAS 2-13) (Fig. 1a and b). Physiological characterization revealed optimal growth conditions for CAAS 2-6 at 25–40°C, pH 7.0–7.5, and 0–4.0% (wt/vol) NaCl tolerance (range: 15–40°C, pH 6.0–8.0, and 0–6.0% NaCl), and for CAAS 2-13 at 25–37°C, pH 5.0–7.5, and 0–3.0% NaCl (range: 15–40°C, pH 4.5–8.0, and 0–5.0% NaCl), with supporting data provided in Fig. 1c. Both strains formed circular, smooth-surfaced colonies with entire margins and convex elevation on LB agar, measuring 0.5–1.0 mm (CAAS 2-6) and 1.5–2.0 mm (CAAS 2-13) in diameter (Fig. S1a). Analysis of the growth curves revealed distinct physiological traits for each strain (Fig. S1b). Strain CAAS 2-13 entered the exponential phase more rapidly and sustained a steeper growth slope than CAAS 2-6. Calculation of the growth parameters confirmed this observation: the maximum specific growth rate (*μ*_max_) for CAAS 2-13 was 0.48 h^−1^, which corresponds to a generation time of 1.44 h. In comparison, strain CAAS 2-6 displayed a higher *μ*_max_ of 0.95 h^−1^ and a shorter generation time of 0.73 h.

Both CAAS 2-6 and CAAS 2-13 exhibit oxidase-negative and catalase-positive. Comparative phenotypic analysis of strains CAAS 2-6, CAAS 2-13, and *A. gerneri* KCTC 12415^T^ is presented in Table S2. API ZYM/20E/20NE/50CH profiles confirmed these strains as members of the genus *Acinetobacter*. Consistent with the genus-specific enzymatic profile, neither strain produced arginine dihydrolase, lysine decarboxylase, ornithine decarboxylase, trypsin, chymotrypsin, α-galactosidase, β-galactosidase, α-glucosidase, β-glucosidase, β-glucuronidase, N-acetyl-β-glucosaminidase, α-mannosidase, or β-fucosidase. However, both exhibited activity for alkaline phosphomonoesterase, acid phosphomonoesterase, esterase (C4), esterase lipase (C8), and leucine arylamidase. Distinctive features included: (i) CAAS 2-6 exhibited gelatinase negativity coupled with atypical metabolic capabilities including acid production from multiple substrates and assimilation of mannose, sucrose, and starch—characteristics divergent from all validated *Acinetobacter* spp.; (ii) CAAS 2-13 demonstrated restricted catabolic competence, showing weak acidification of sorbitol, d-mannitol, and sucrose, along with failure to assimilate d-glucose, AA, or utilize various carbon sources, thereby differentiating it from other members of the genus.

The whole-cell fatty acid profiles of strains CAAS 2-6, CAAS 2-13 and *A. gerneri* KCTC 12415^T^ are presented in Table 1. Strain CAAS 2-6 contained Summed Feature 3 (C_16:1_ *ω*7*c* and/or C_16:1_ *ω*6*c*, 25.8%), C_16:0_ (25.7%), and C_18:1_ *ω*9*c* (10.5%) as major components, while strain CAAS 2-13 showed predominant levels of Summed Feature 3 (24.1%), C_16:0_ (24.5%), and C_18:1_ *ω*9*c* (21.6%). The polar lipid profiles of strain CAAS 2-6 and *A. gerneri* KCTC 12415^T^ comprised diphosphatidylglycerol (DPG), phosphatidylethanolamine (PE), phosphatidylglycerol (PG), aminolipid, unidentified phosphoglycolipids, aminophospholipids, unknown polar lipids (L) (Fig. S2). These chemotaxonomic characteristics aligned with those of recently described *Acinetobacter* species (24, 35, 81), confirming the phylogenetic placement of both strains within the genus *Acinetobacter*.

**TABLE 1:** Fatty acid results of strains (1) CAAS 2-6, (2) CAAS 2-13, and (3) *A. gerneri* KCTC 12415^T*a*^

Fatty acid	1	2	3
C_10:0_	tr	tr	tr
Iso-C_12:0_	tr	−	−
C_12:0_	7.0	8.7	9.6
Iso-C_13:0_	1.8	tr	tr
Anteiso-C_13:0_	0.6	−	tr
C_12:0_ 2-OH	0.5	0.9	8.9
C_12:0_ 3-OH	3.4	5.3	**13.2**
Iso-C_14:0_	1.3	−	−
C_14:0_	1.2	0.5	0.8
Iso-C_15:0_	3.8	−	−
Anteiso-C_15:0_	1.3	−	tr
C_16:1_ *ω*7*c* alcohol	1.8	−	−
C_16:0_ N alcohol	6.5	3.8	−
Iso-C_16:0_	1.6	−	−
C_16:0_	**25.7**	**24.5**	**17.7**
Iso-C_17:1_ *ω*10*c*	tr	−	tr
Iso-C_17:0_	1.1	tr	tr
C_17:1_ *ω*8*c*	tr	0.6	tr
C_17:0_	tr	0.5	tr
C_18:3_ *ω*6*c* (6, 9, 12)	2.1	3.1	tr
C_18:1_ *ω*9*c*	**10.5**	**21.6**	**30.3**
C_18:0_	1.1	2.4	2.0
C_19:0_ cyclo *ω*8*c*	−	−	−
Summed Feature 2	0.5	tr	2.9
Summed Feature 3	**25.8**	**24.1**	**12.1**
Summed Feature 8	0.6	2.8	−

^*a*^ Summed Feature 2 comprised C_12:0_ aldehyde ?, Summed Feature 3 comprised C_16:1_ *ω*7*c *and/or C_16:1_ *ω*6*c*, Summed Feature 5 comprised C_18:2_ *ω*6, 9*c *and/or Ante-C_18:0_, Summed Feature 8 comprised C_18:1_ *ω*7*c*. –, not detected, less than 0.5% as tr. Fatty acids present at >10 % are highlighted in bold. Data are presented to two decimal places.

**Fig 2:**
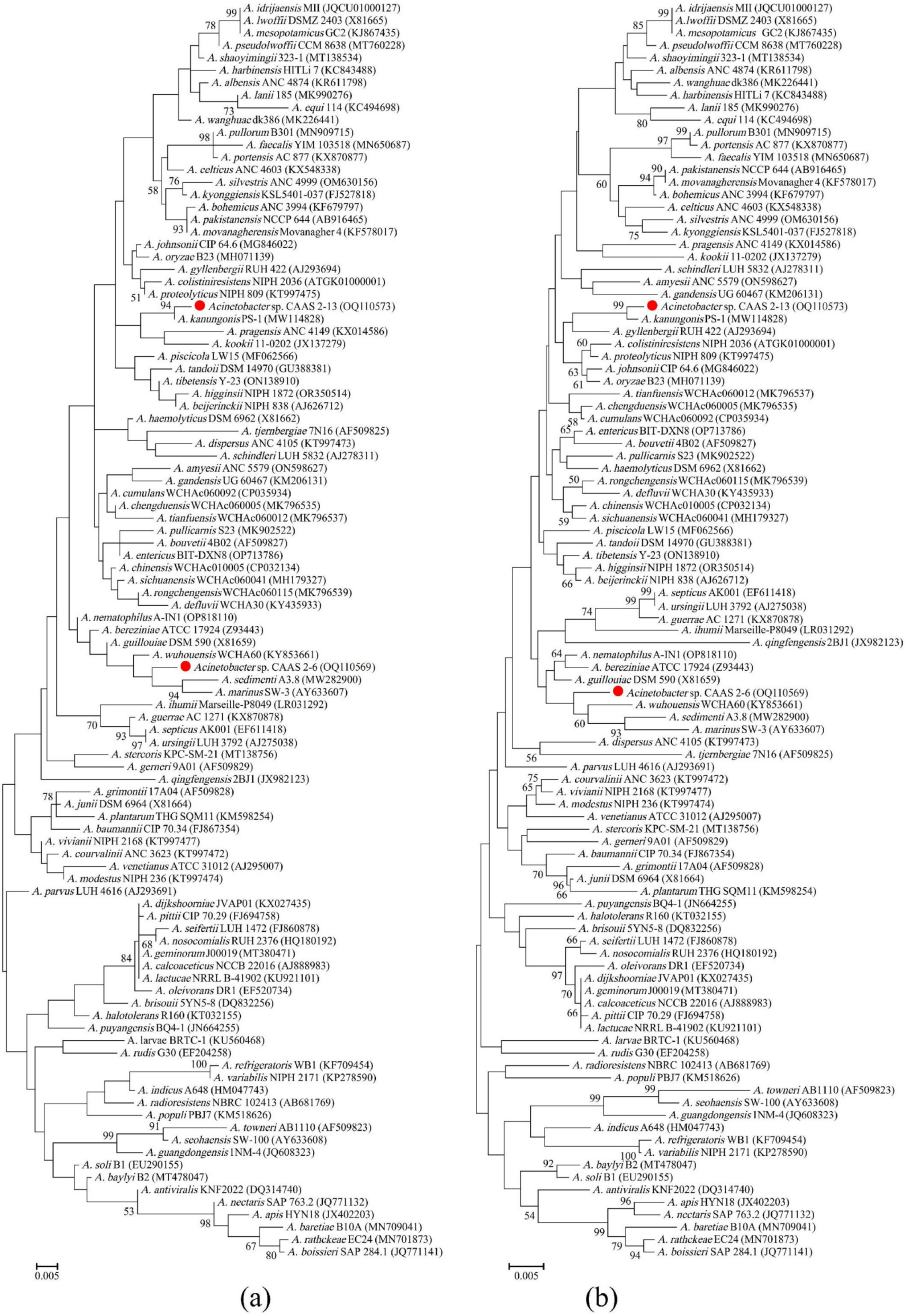
The ML (**a**) and NJ (**b**) phylogenetic trees were constructed based on *16S rRNA* gene sequences of strains CAAS 2-6 and CAAS 2-13, along with 96 type strains of validly published *Acinetobacter* species. Bootstrap values (expressed as percentages) were calculated from 1,000 replicates, with values below 50% omitted for clarity. A scale bar indicating 0.01 nucleotide substitutions per site is shown.

Phylogenetic analysis based on *16S rRNA* gene sequences indicated that strain CAAS 2-6 (OQ110573) shared the highest similarity with *A. gerneri* DSM 14967^T^ (97.7%), followed by *A. kanungonis* PS-1^T^ (97.6%) and *A. tandoii* DSM 14970^T^ (97.5%). In contrast, strain CAAS 2-13 (OQ110569) showed 99.6% similarity with *A. kanungonis* PS-1^T^, while its similarity to other recognized *Acinetobacter* species did not exceed 98.5%. The evolutionary relationships were further elucidated by both ML and NJ phylogenetic trees, which included 96 type strains of validly published *Acinetobacter* species (Fig. 2). In these trees, strain CAAS 2-6 formed a distinct phylogenetic branch, supporting its status as a novel species. Although strain CAAS 2-13 clustered closely with *A. kanungonis* PS-1^T^ with robust bootstrap support (>90%), the observed genetic divergence suggests that it may represent a novel subspecies. Several additional well-supported clades were also identified, including: (i) *A. idrijacnsis* Mll^T^, *A. lwoffii* DSMZ 2403^T^, and *A. mesopotamicus* GC2^T^; (ii) *A. pakistanensis* NCCP 644^T^ and *A. movanagherensis* Movanagher 4^T^; (iii) *A. septicus* AKO01^T^ and *A. uusingii* LUH 3792^T^; and (iv) *A. dijkshoorniae* JVAP01^T^, *A. geminorum* J00019^T^, *A. calcoaceticus* NCB 22016^T^, *A. pitti* CIP 70.29^T^, and *A. lactucae* NRRL B-41902^T^. Collectively, these phylogenetic results robustly support the designation of CAAS 2-6 as a novel species within the genus *Acinetobacter* and suggest that CAAS 2-13 likely represents a novel subspecies of *A. kanungonis*.

### Genomic features and analyses

**Fig 3:**
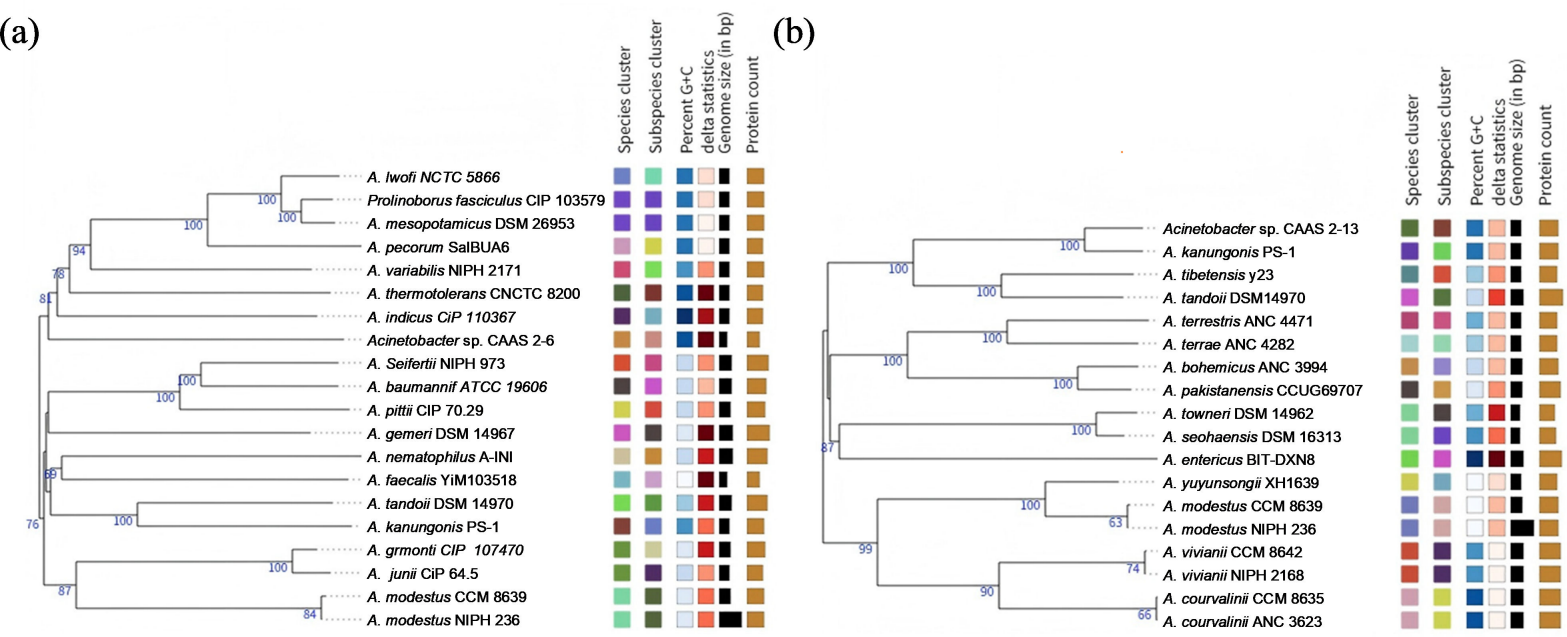
Phylogenomic tree of strains CAAS 2-6 (**a**) and CAAS 2-13 (**b**) inferred from TYGS analysis.

The draft genome of strain CAAS 2-6 was sequenced to a coverage of 469×, yielding 1.236 Gb of raw data assembled into 73 scaffolds. The genome size was 2.59 Mb with a G + C content of 44.4%, encoding 2,344 genes, 59 tRNAs, 3 rRNAs, and no CRISPR arrays. Functional annotation assigned 2,344, 2,089, 1,783, and 1,803 genes to the NR, eggNOG, KEGG, and Swiss-Prot databases, respectively. The genome sequence has been deposited under GenBank accession number JAQIHG000000000 (Table S2). Similarly, strain CAAS 2-13 was sequenced to 308× coverage (1.113 Gb raw data), assembled into 48 scaffolds totaling 3.49 Mb with a G + C content of 42.0%. It contained 3,332 genes, 66 tRNAs, 10 rRNAs, and no CRISPRs, with 3,273, 2,841, 1,773, and 2,317 genes annotated in the NR, eggNOG, KEGG, and Swiss-Prot databases, respectively (GenBank: JAQIHH000000000; Table S2). Phylogenomic analysis via TYGS delineated the taxonomic positions of strains CAAS 2-6 and CAAS 2-13 (Fig. 3). CAAS 2-6 formed a distinct lineage, with genomic relatedness to its closest relative, *A. indicus* CIP 110367^T^, falling well below established species thresholds (dDDH: 19.7–22.7%; ANI: 74.5–77.0%; ANIb: 72.0–76.8%; and OrthoANI: 74.3–76.8%; Table S3; Fig. S3a and c). In contrast, CAAS 2-13 clustered closely with *A. kanungonis* PS-1^T^ but was genetically distinct, showing relatedness values (dDDH: 66.5%; ANI: 96.0%; ANIb: 95.6%; and OrthoANI: 96.0%; Table S3; Fig. S3b and d) that support subspecies-level divergence. Based on these results and adhering to standard thresholds for species (70% dDDH and 95–96% ANI) (82) and subspecies (≤79% dDDH and ≈97.7% ANI) (83) delineation, we propose CAAS 2-6 as a novel species within the genus *Acinetobacter* and CAAS 2-13 as a novel subspecies of *A. kanungonis*.

**Fig 4:**
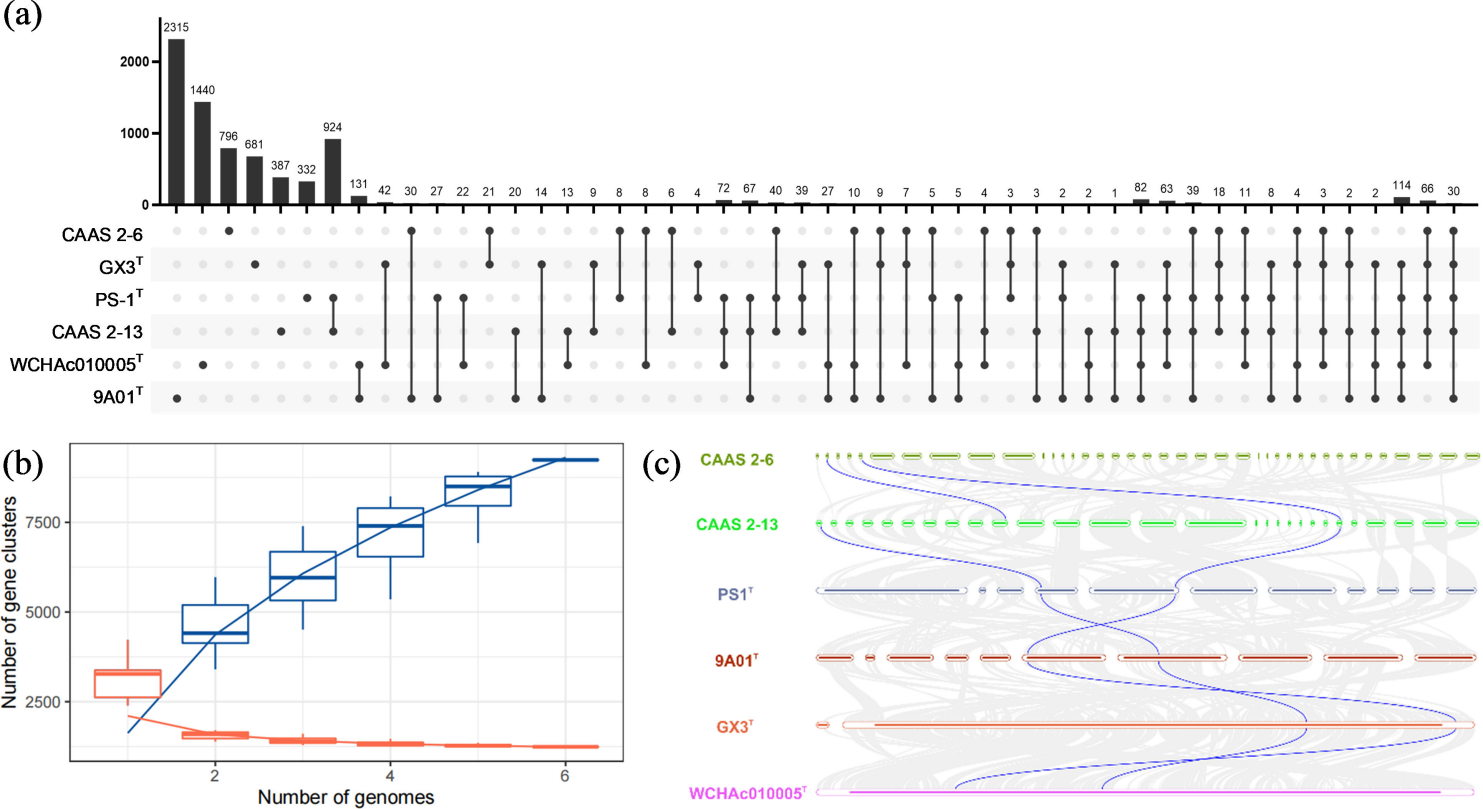
Pan-genome and genomic collinearity analysis of six *Acinetobacter* strains. (**a**) Upset plot illustrating the distribution of gene clusters across the analyzed strains. The vertical bars represent the size of gene clusters shared by the combination of strains indicated by the connected dots below. (**b**) Core-pan rarefaction curve. The pan-genome size (total gene repertoire) and core-genome size (genes shared by all strains) are plotted as a function of the number of sequentially added genomes. (**c**) Genomic collinearity analysis. Syntenic blocks between the six genomes are connected by colored lines, with each strain assigned a specific color**:** *Acinetobacter* sp. CAAS 2-6 (dark green), *Acinetobacter* sp. CAAS 2-13 (bright green), *A. kanungonis* PS1^T^ (dark blue), *A. gerneri* 9A01^T^ (brown), *A. towneri* GX3^T^ (orange), and *A. chinensis* WCHAc010005^T^ (pink). Key genes encoding multicopper oxidase (AbMCO) and alkane hydroxylase (AlkB) are highlighted by blue connecting lines, indicating conserved synteny.

The pan-genome analysis of six strains within the genus *Acinetobacter*, including CAAS 2-6, CAAS 2-13, *A. chinensis* WCHAc010005^T^, *A. towneri* GX3^T^, *A. gerneri* 9A01^T^, and *A. kanungonis* PS1^T^, revealed remarkably open genomic characteristics (Fig. 4a and b). The distribution of gene clusters in the Upset plot showed a predominance of strain-specific genes, the largest of which were found in *A. gerneri* 9A01^T^ (2,315 genes) and *A. chinensis* WCHAc010005^T^ (1,440 genes). A strikingly large, exclusive cluster of 924 genes was shared only between CAAS 2-13 and *A. kanungonis* PS1^T^. This unique and substantial shared gene content, which far surpassed that of any other pairwise combination, provides strong evidence for a close evolutionary relationship between these two strains. The core genome analysis identified 1,482 core gene families, which constitute the essential genetic foundation required for survival across these strains. In stark contrast, the pan-genome size reached 92,340 gene families, a scale that vastly exceeds that of the core genome, indicating an exceptionally high level of genetic diversity. Most critically, the pan-genome rarefaction curve exhibited a typical open pattern; as the number of sequenced strains increased, the discovery of new gene families showed no sign of saturation but continued to grow steadily and stably. This result demonstrates that the genus *Acinetobacter* possesses a massive gene reservoir that remains largely unexplored. This open pan-genome structure serves as the genetic basis for its powerful environmental adaptability, whereby different strains, including these type strains, acquire a substantial number of unique accessory and strain-specific genes to adapt to diverse ecological niches. Comparative synteny analysis revealed distinct patterns of genome conservation and rearrangement among the six strains (Fig. 4c). CAAS 2-13 and *A. kanungonis* PS-1^T^ shared extended, contiguous syntenic blocks, indicating strong conservation of gene order. Conversely, the other four strains (CAAS 2-6, *A. chinensis* WCHAc010005^T^, *A. towneri* GX3^T^, and *A. gerneri* 9A01^T^) showed shorter and more fragmented collinear blocks, indicating a greater incidence of genome rearrangements. In line with the pan-genome results, the synteny analysis collectively underscores the close phylogenetic relationship between CAAS 2-13 and *A. kanungonis* PS-1^T^, while highlighting the greater evolutionary divergence and genomic restructuring in the other strains.

### Plastic biodegradation and growth capacity of diverse *Acinetobacter* strains

**Fig 5:**
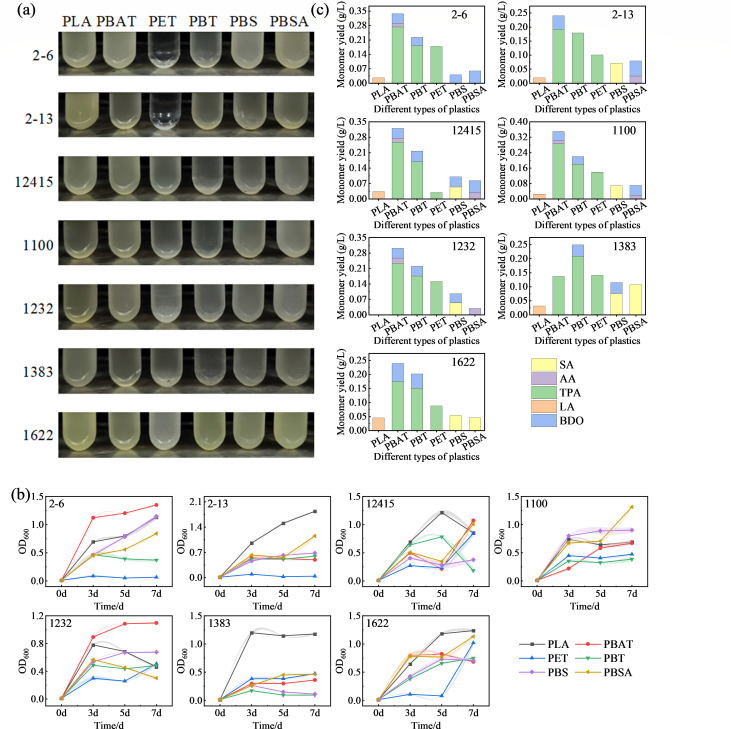
Substrate-dependent growth of *Acinetobacter* strains on synthetic polyesters. (**a**) Representative photographs of bacterial cultures after 7 days of incubation. (**b**) Growth kinetics monitored by OD_600_, data points represent the mean ± SD (*n* = 3). (**c**) Quantification of released monomers in the culture supernatant by HPLC. Values are the mean ± SD of three biological replicates. Statistical analysis was performed using one-way ANOVA followed by Tukey’s post hoc test (*P* < 0.05). Different lowercase letters above the bars indicate statistically significant differences among the groups.

Type strains *A. gerneri* 9A01^T^ = KCTC 12415^T^ (abbreviated 12,415), *A. wuhouensis* WCHA60^T^ = GDMCC 1.1100^T^ (1,100), *A. chinensis* WCHAc010005^T^ = GDMCC 1.1232^T^ (1,232), *A. sichuanensis* WCHAc060041^T^ = GDMCC 1.1383^T^ (1,383), and *A. chengduensis* WCHAc060005^T^ = GDMCC 1.1622^T^ (1,622) served as reference controls alongside target isolates CAAS 2-6 and CAAS 2-13 in plastic degradation assays. After a 7-day incubation in modified MSM with single plastic substrates (PLA, PBAT, PET, PBT, PBS, or PBSA), turbidity measurements revealed differential growth capacities (Fig. 5a). Both CAAS 2-6 and CAAS 2-13 showed no observable growth in PET-amended medium, while *A. sichuanensis* GDMCC 1.1383^T^ exhibited consistently poor growth across all plastics. Significantly enhanced growth was observed in aliphatic polyesters (PLA, PBAT, PBS, and PBSA) compared to aromatic polyesters (PET and PBT), aligning with the established recalcitrance of aromatic polyesters to biodegradation. Degradation efficiency ranking indicated: CAAS isolates > four reference strains > *A. sichuanensis* GDMCC 1.1383^T^.

Plastic degradation capacities of seven *Acinetobacter* strains were systematically evaluated by monitoring growth kinetics (OD_600_) and monomer yields across six polyester substrates: PLA, PBAT, PET, PBT, PBS, and PBSA (Fig. 5b and c). The results demonstrated that strain CAAS 2-6 exhibited optimal PLA-driven growth (OD_600_ = 1.13 ± 0.04) attributed to efficient lactate metabolism, correlating with low lactate accumulation (0.03 g/L). Peak biomass occurred on PBAT (OD_600_ = 1.35 ± 0.01), with a TPA yield of 0.27 g/L. This yield was the highest among terephthalate-containing substrates, in the order of PBAT > PBT > PET. Strain CAAS 2-13 demonstrated maximal PLA growth (OD_600_ = 1.84 ± 0.02) with minimal lactate accumulation (<0.02 g/L). TPA production declined progressively: PBAT (0.19 g/L) > PBT (0.18 g/L) > PET (0.10 g/L). PBS degradation yielded SA (0.07 g/L), while PBSA degradation produced both AA (0.03 g/L) and DBO (0.05 g/L). Five reference strains exhibited habitat-independent degradation capacities (OD_600_ = 0.19–1.31), confirming genus-wide plastic-degrading potential. These findings demonstrate that habitat-driven adaptive evolution confers substrate-specific polyester degradation capabilities in *Acinetobacter*, as evidenced by: (i) specialized aliphatic polyester (PLA/PBS/PBSA) degradation in CAAS strains; (ii) convergent evolutionary adaptation enhancing co-habitat strain efficiency; and (iii) genus-wide ecological versatility enabling synthetic polyester degradation. This work establishes *Acinetobacter* as a prime candidate for enzymatic plastic upcycling, with significant bioremediation potential in environmental biotechnology.

**Fig 6:**
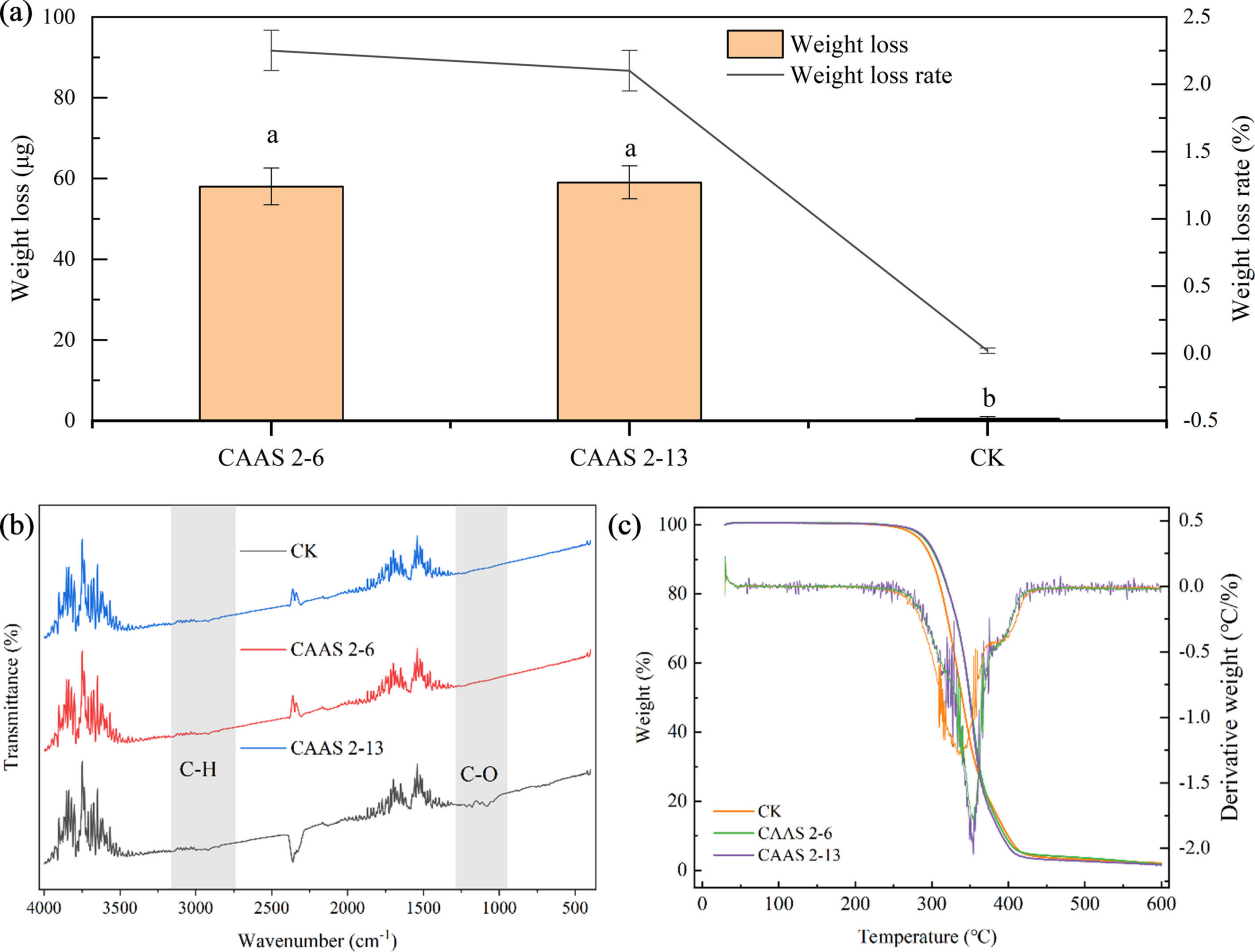
Physicochemical characterization of PLA films after microbial degradation. (**a**) FTIR spectra and (**b**) TG analysis (left *Y*-axis) with corresponding DTG curves (right *Y*-axis) of PLA films incubated with strain CAAS 2-6, strain CAAS 2-13, and an uninoculated control (CK).

Strains CAAS 2-6 and CAAS 2-13 demonstrated significant degradation activity against PLA film, with weight losses of 58.00 ± 4.55 and 59.00 ± 4.08 μg, corresponding to degradation rates of 2.25 ± 0.15% and 2.10 ± 0.15%, respectively. The comparable weight loss and degradation rates between the two strains suggest consistent and efficient enzymatic depolymerization of PLA. The degradation of PLA by strains CAAS 2-6 and CAAS 2-13 was highly selective, primarily targeting the ester bond backbone of the polymer while leaving side-chain groups, such as methyl groups, largely unaffected. This specificity is evidenced by FTIR spectroscopy (Fig. 6a), which showed a significant attenuation of absorption bands in the 1,000–1,250 cm^−1^ region, corresponding to the C–O–C and C–O stretching vibrations of the ester linkages. This indicates enzymatic scission of the polymer main chain. In contrast, the C–H stretching vibration bands in the 2,750–3,000 cm^−1^ region remained virtually unchanged, confirming the structural integrity of the methyl side chains. TG and DTG analysis further revealed that microbial degradation selectively altered the thermal properties of PLA (Fig. 6b). The delayed onset of weight loss in the treated films suggests that the strains preferentially consumed amorphous or thermally less stable regions, thereby enriching the residue with more recalcitrant, crystalline components. This led to an initial increase in thermal stability. Subsequently, these “concentrated” recalcitrant components underwent rapid decomposition at higher temperatures, manifesting as a higher maximum rate of weight loss. The slightly greater degradation rate observed for CAAS 2-13, compared to CAAS 2-6, points to subtle differences in enzymatic activity or metabolic specificity, resulting in marginally more efficient depolymerization.

### Expression and enzymatic degradation, and structural analysis of multicopper oxidase AbMCO and alkane hydroxylase AlkB

Based on genomic data, we cloned the coding sequences of multicopper oxidase AbMCO and alkane hydroxylase AlkB for heterologous expression in the pET-28a(+) system. Recombinant proteins from various *Acinetobacter* strains were successfully expressed in *E. coli* BL21(DE3) under the control of the *lac* promoter with N-terminal His_6_-tags. Protein samples from recombinant *E. coli* (whole-cell lysates, soluble fractions, and inclusion bodies) were analyzed by SDS-PAGE (12% separating gel). Each recombinant protein migrated as a predominant single band (Fig. S4). Calculated molecular masses excluding the His-tag are approximately 70.8 kDa for 2-6AbMCO1, 66.8 kDa for 2-13AbMCO 1, 69.4 kDa for 2-13AbMCO 2, 66.1 kDa for 12415AbMCO, 67.4 kDa for 1100AbMCO, 65.4 kDa for 1232AbMCO, 63.1 kDa for 1383AbMCO, 66.7 kDa for 1622AbMCO, 46.2 kDa for 2-13AlkB, and 46.2 kDa for 1100AlkB.

**Fig 7:**
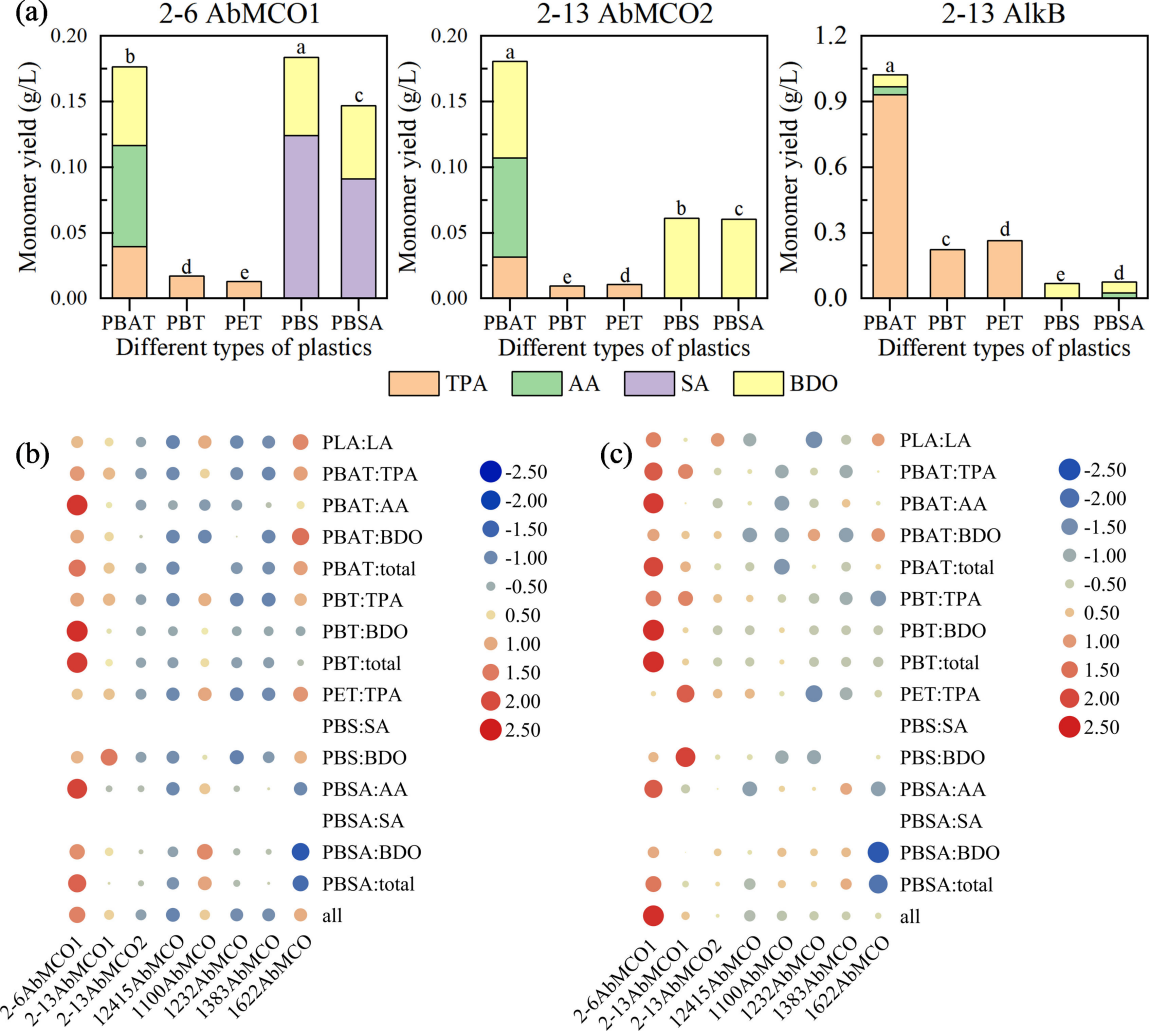
Enzymatic depolymerization of plastics by heterologously expressed AbMCO and AlkB. (**a**) Representative HPLC chromatograms of degradation products after incubation of polymers with the purified enzymes. Control reactions contained no enzyme. Bars represent the mean ± SD (*n*=3), and statistical significance is indicated as in Fig. 5. (**b**) Heat map representing the concentration of the primary monomeric product (g/L) released in the validation assay. (**c**) Heat map of the enzymatic yield (g/g), calculated as grams of product per gram of polymer substrate.

Due to inclusion body formation by recombinant alkane hydroxylase AlkB, refolding was performed with urea according to the HyPur T Ni-NTA 6FF Gravity Column protocol (Sangon Biotech, C600791-0010), followed by affinity purification using Ni-NTA resin. Initial purification targeted soluble fractions of 2-6AbMCO1 and 2-13AbMCO2, along with refolded soluble fractions from 2-13AlkB. Purified recombinant enzymes (200 μL) were incubated with 800 μL of reaction buffer and polyester substrates (0.2 g/L powder, <100 µm particle size) at 37°C with agitation (180 rpm) for 120 h. After incubation, the supernatants were analyzed by HPLC. For the validation assays, rapid protein purification was conducted at 4°C, followed by the initiation of enzymatic depolymerization initiated within 24 h. HPLC analysis (Fig. 7) revealed higher monomer yields from AlkB-mediated degradation versus AbMCO, particularly for TPA from PBAT (>0.9 g/L; Fig. 7a). Among all AbMCO variants, 2-6AbMCO1 (Fig. S6) demonstrated optimal catalytic performance: total product yield of 3.8 g/L with enzymatic productivity of 9.4 g product/g enzyme. In contrast, 12415AbMCO showed significantly reduced efficiency (Fig. 7b and c).

To further elucidate the product profiles of six plastics depolymerized by the enzyme 2-6AbMCO1, the enzymatic hydrolysates were subjected to LC-MS analysis (Fig. S7). The results demonstrated distinct degradation susceptibilities among the polymers: PBT and PET exhibited minimal degradation, as evidenced by the absence of prominent product peaks in the total ion current chromatograms. In contrast, analysis of mass spectra at specific scan points for PLA, PBAT, PBS, and PBSA revealed detectable oligomeric fragments with significant ion intensity, indicating active depolymerization. Among the top 20 metabolites ranked by relative abundance, the LA dimer (C_6_H_12_O_6_) derived from PLA was notably predominant, distinguishing it from the other five plastics. Additionally, AA monomer was most abundantly produced in PBAT, though it ranked 20th overall. Based on the extent of degradation observed, the enzymatic degradation efficiency of 2-6AbMCO1 toward the six plastics can be ordered as follows: PLA > PBAT > PBS > PBSA > PBT > PET. This hierarchy suggests that the enzyme has a higher affinity for aliphatic polyesters like PLA and PBSA, while aromatic polyesters such as PBT and PET remain more recalcitrant, likely due to their crystalline structures and stable aromatic bonds.

Multiple sequence alignment of multicopper oxidase AbMCO and alkane hydroxylase AlkB (Fig. S6) revealed divergent conservation patterns. AbMCO exhibited poor conservation in mid-rear and C-terminal regions, correlating with predicted random coil structures. The multicopper oxidase AbMCO, which is highly conserved across *A. baumannii* strains, exhibits *in vitro* enzymatic activity only upon supplementation with ≥1 μM copper ions, demonstrating its obligate copper dependence (84). AlkB showed low sequence conservation at N-terminal, mid-rear, and C-terminal segments, while conserved domains predominantly adopted α-helical conformations. Despite significant sequence diversity, AlkB enzymes consistently catalyze the initial hydroxylation of hydrocarbons, with conserved structural features in medium-length alkane biodegradation (85).

**Fig 8:**
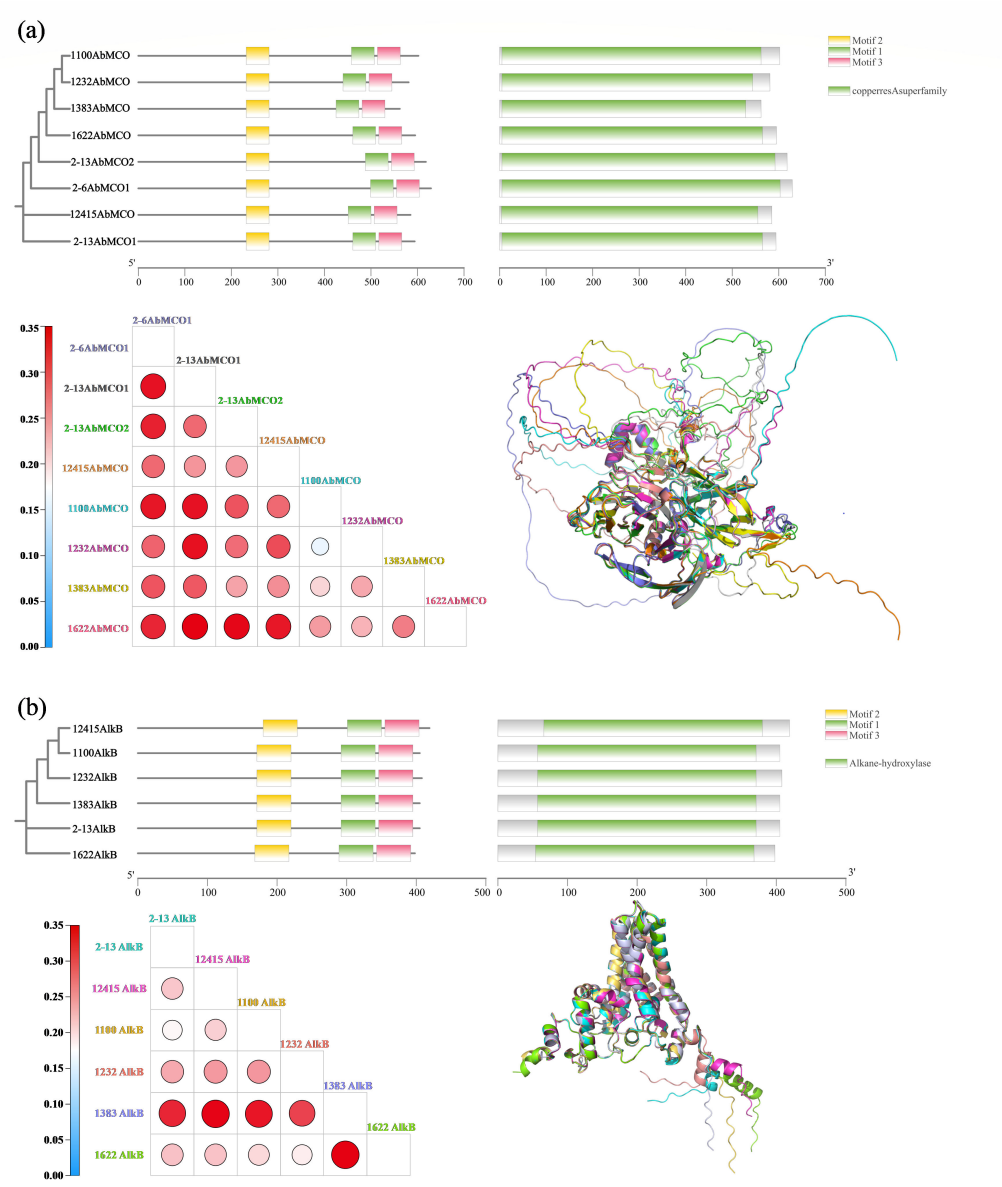
Comparative analysis of evolutionary relationships and structural features among recombinant AbMCO (**a**) and AlkB (**b**) from various *Acinetobacter* strains. Top: Phylogenetic tree based on amino acid sequences. Bottom left: The RMSD values (in Å) of the aligned AlphaFold-predicted model structures, indicating the similarity between protein structures. Bottom right: Visual representations of the structural alignment results. The color of protein labels in the phylogenetic tree corresponds to the color of the same proteins in the structural comparison visualizations.

Phylogenetic analysis placed 12415AbMCO and 2-13AbMCO1 on basal branches with significant genetic divergence. A highly supported clade (Bootstrap = 97–100) comprising 1100AbMCO, 1232AbMCO, 1383AbMCO, and 1622AbMCO displayed short branch lengths, indicating strong sequence conservation. 2-13AlkB and 1622AlkB occupied distal positions, while the ancestral node of 12415AlkB-1383AlkB showed moderate support (Bootstrap = 64–87), warranting further validation. AlphaFold structural comparisons yielded low root-mean-square deviation (RMSD) values among 1100AbMCO, 1232AbMCO, 1383AbMCO, and 1622AbMCO, confirming high structural similarity. Despite shared ecological origins, 2-6AbMCO1 diverged structurally from 2 to 13AbMCO1/2-13AbMCO2. 1383AlkB showed marked structural deviation from other AlkBs, even though 2-13AlkB was phylogenetically distant (Fig. 8).

Structural alignments of AlphaFold-predicted AbMCO models revealed a predominantly β-sheet-rich fold, characterized by minimal α-helical content and extensive random coil regions. A disordered segment within this structure correlates with low prediction confidence, likely attributable to limited homology with experimentally resolved templates. Notably, its substrate-binding domain forms a negatively charged catalytic pocket, where hydrophobic residues line the binding site and substrate access is regulated by rigid α-helical gatekeeping elements. Conversely, AlkB adopts a scaffold-like architecture typical of non-heme diiron monooxygenases. This structure comprises: (i) six transmembrane helices (TM1–TM6) constituting a substrate-access channel, (ii) an N-terminal cytosolic catalytic domain housing a hydrophilic di-iron center essential for oxygen activation, and (iii) a membrane-embedded C-terminus adjacent to the catalytic domain that stabilizes the complex (Fig. 8).

**Fig 9:**
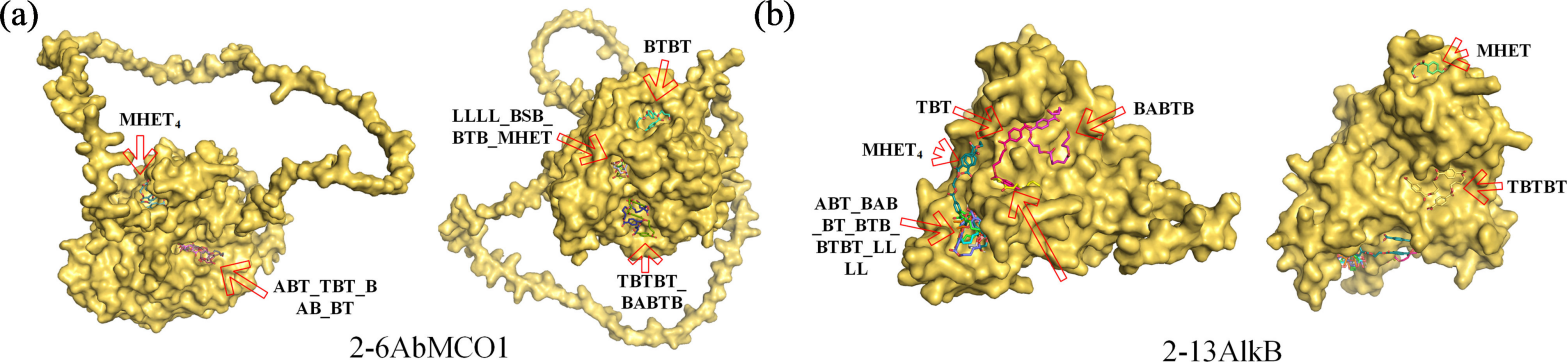
Molecular docking of 2-6AbMCO1 and 2-13AlkB. (**a**) 2-6AbMCO1 binding sites. (**b**) 2-13alkB binding sites.

Based on plastic depolymerization activity and AlphaFold structural alignments, molecular docking of representative enzymes 2-6AbMCO1 and 2-13AlkB with 12 oligomeric substrates (ABT, BAB, BABTB, BSB, BT, BTB, BTBT, LLLL, MHET, MHET4, TBT, and TBTBT) revealed distinct binding topologies: 2-6AbMCO1 accommodated substrates at five binding sites—Site 1 (BSB/BTB/LLLL/MHET), Site 2 (ABT/TBT/BAB/BT), Site 3 (BABTB/TBTBT), and exclusive Sites 4–5 for BTBT and MHET4 respectively; whereas 2-13AlkB utilized seven loci predominantly at its base—including a shared catalytic pocket (Site 1: ABT/BAB/BT/BTB/BTBT/LLLL), vicinal sites for MHET4/BSB/TBT/BABTB (Sites 2–5), a waist-positioned site for TBTBT (Site 6), and an apical site for MHET (Site 7)—notably exhibiting differential residue lining patterns within the common pocket occupied by six substrates (Fig. 9).

### Conclusion

In this study, strains CAAS 2-6 and CAAS 2-13 were isolated from landfill leachate and a strawberry cultivation field, respectively. These novel plastic-degrading strains were taxonomically identified as *A. minutum* sp. nov. and *A. kanungonis* subsp. *fragariae* subsp. nov. Both CAAS 2-6 and CAAS 2-13 alongside five reference strains, were demonstrated the ability to degrade various plastic types. Notably, strains CAAS 2-6 and CAAS 2-13 grew on plastics (PLA, PBAT, PBT, PBS, and PBSA) as the sole carbon source, reaching an OD_600_ of 0.3 within 3–7 days. Genomic mining revealed the expression of the multicopper oxidase AbMCO and the alkane hydroxylase AlkB. Enzymatic depolymerization assays confirming their involvement in plastic degradation. LC-MS analysis confirmed the enzymatic activity of 2-6AbMCO1 through the detection of characteristic oligomers and monomers; the enzyme exhibited a distinct preference, showing significantly higher efficacy against aliphatic polyesters such as PLA compared to aromatic ones. These findings underscore the potential of harnessing *Acinetobacter* species for plastic waste bioremediation and open avenues for synthetic biology approaches to upcycle plastic waste into valuable chemicals. Future work should prioritize purification and biochemical characterization of specific extracellular esterases (e.g., EC 3.1 hydrolases), leverage machine learning tools to analyze genomic data, and evaluate performance in real-world environments.

### Description of *A. minutum* sp. nov.

*A. minutum* (mi.nu’tum. L. neut. adj. minutum, very small, minute, referring to the diminutive size of the colonies and the low cell biomass yield).

The strain CAAS 2-6^T^, isolated from landfill leachate, forms circular, regular-edged, smooth colonies of 0.5–1.0 mm in diameter on LB plates after being incubated at 30°C for 2 days. The cells are rod-shaped, measuring 0.6–0.7 µm × 0.7–1.3 µm. The strain grows within a temperature range of 15–40°C, with an optimum growth temperature of 25–40°C. It can grow in the presence of 0–6.0% (wt/vol) NaCl, with optimal growth observed at 0–4.0% (wt/vol) NaCl. The pH range for growth is 6.0–8.0, with an optimum pH of 7.0–7.5. *16S rRNA* sequence (GenBank accession number: OQ110573) analysis revealed a similarity of 97.7%, 97.6%, 97.5%, and 97.5% with *A. gerneri* DSM 14967^T^, *A. kanungonis* PS-1^T^, *A. tandoii* DSM 14970^T^ and *A. junii* CIP 64.5^T^, respectively. Evolutionary tree analysis based on *16S rRNA* sequences, as well as the housekeeping genes *gyrB* and *rpoB*, showed that CAAS 2-6^T^ formed a separate cluster. The strain is negative for oxidase, gelatinase, and nitrate reduction and cannot assimilate AA, malic acid, and sodium citrate. It cannot utilize l-arabinose, d-xylose, galactose, and amygdalin. However, it tests positive for the Voges-Proskauer reaction, can assimilate d-glucose, and can ferment d-glucose, d-mannitol, inositol, sorbitol, rhamnose, sucrose, melibiose, amygdalin, and l-arabinose to produce acid. It can also utilize glucose, fructose, mannose, N-acetyl-glucosamine, amygdalin, arbutin, esculin, maltose, sucrose, and starch. The G + C content of the genome (GenBank accession number: JAQIHG000000000/GCA_035916735.1) is 44.4 mol%. The highest dDDH and ANI values were observed with *A. indicus* CIP 110367^T^, at 22.7% and 77.0%, respectively. The main fatty acids are Summed Feature 3 (comprising C16:1 *ω*7c and/or C_16:1_ *ω*6*c*, 25.8%), C_16:0_ (25.7%), and C_18:1_ *ω*9*c* (10.5%). The major polar lipids are DPG, PE, and PG. Based on the experimental results of physiological and biochemical characteristics, genomic homology, and content, the CAAS 2-6^T^ strain is considered a new species of the genus *Acinetobacter*. Due to its distinguishing growth rate compared to other species within the genus, it has been named *A. minutum* sp. nov. The CAAS 2-6^T^ strain, as the type strain of *A. minutum*, has been preserved at the China General Microbiological Culture Collection Center, the Japan Collection of Microorganisms, and the Korean Collection for Type Cultures with preservation numbers GDMCC 1.3951, JCM 36321, and KCTC 8156, respectively.

### Description of *A. kanungonis* subsp. *fragariae* subsp. nov.

*A. kanungonis* subsp. *fragariae* (frag’ar.i.ae. N.L. fem. n. Fragaria, generic name of strawberry; N.L. gen. fem. n. fragariae, of Fragaria).

The strain CAAS 2-13^T^, isolated from a strawberry field, forms circular, regular-edged, smooth, and flat colonies of 1.5–2.0 mm in diameter on LB plates after being incubated at 30°C for 2 days. The cells are short rod-shaped, with dimensions of 0.5–0.6 µm × 0.7–1.2 µm. This strain grows within a temperature range of 15–40°C, with an optimum growth temperature of 20–37°C. It can grow in the presence of 0–5.0% (wt/vol) NaCl, with optimal growth observed at 0–3.0% (wt/vol) NaCl. The pH range for growth is 4.5–8.0, with the most suitable pH being 5.0–7.5. Analysis of the *16S rRNA* sequence (GenBank accession number: OQ110569) revealed a high similarity of 99.6% to the *16S rRNA* sequence of *A. kanungonis* PS-1^T^. The *16S rRNA* sequence-based phylogenetic tree clustering is closest to *A. kanungonis* PS-1^T^, while the phylogenetic clustering based on the housekeeping genes *gyrB* and *rpoB* is closest to *A. tandoii* CIP 1074697^T^. The strain is negative for oxidase, Voges-Proskauer reaction, and nitrate reduction. It cannot ferment d-glucose, inositol, sorbitol, rhamnose, melibiose, amygdalin, or l-arabinose to produce acid, and it cannot assimilate d-glucose or AA. Additionally, it cannot utilize l-arabinose, ribose, d-xylose, galactose, mannose, amygdalin, arbutin, sucrose, or starch. However, it is gelatinase-positive, can assimilate malic acid and sodium citrate, and can utilize glucose, fructose, N-acetyl-glucosamine, esculin, and maltose. The G + C content of the genome (GenBank accession number: JAQIHH000000000) is 42.0 mol%. The highest values of dDDH and ANI with *A. kanungonis* PS-1^T^ are 66.5% and 96.0%, respectively. The major fatty acids are C_16:0_ (25.5%), Summed Feature 3 (comprising C_16:1_ *ω*7*c* and/or C_16:1_ *ω*6*c*, 24.1%), and C_18:1_ *ω*9*c* (21.6%). Based on a comprehensive evaluation of physiological and biochemical characteristics, genomic homology, and content, despite the similarity and ANI of the *16S rRNA* sequence being above the recommended thresholds, the dDDH is slightly below the recommended cutoff value. Furthermore, there is a certain evolutionary distance between TYGS and *A. kanungonis* PS-1^T^ in the genomic phylogenetic tree. Therefore, the strain CAAS 2-13^T^ is identified as a novel subspecies of *A. kanungonis* PS-1^T^. Given its distinct isolation source from *A. kanungonis* PS-1^T^, it is named *A. kanungonis* subsp. *fragariae* subsp. nov. The strain CAAS 2-13^T^, representing *A. kanungonis* subsp. *fragariae*, has been preserved at the China General Microbiological Culture Collection Center, the Japan Collection of Microorganisms, and the Korean Collection for Type Cultures with preservation numbers GDMCC 1.3956, JCM 36322, and KCTC 8157, respectively.

## Data Availability

The GenBank/EMBL/DDBJ accession numbers for the genome and *16S rRNA* gene sequences of strain *Acinetobacter minutum* CAAS 2-6^T^ are JAQIHG000000000/GCA_035916735.1 and OQ110573, respectively. The GenBank/EMBL/DDBJ accession numbers for the genome and *16S rRNA* gene sequences of strain *Acinetobacter kanungonis* subsp. *fragariae* CAAS 2-13^T^ are JAQIHH000000000 and OQ110569, respectively.

## SUPPLEMENTAL MATERIAL

Supplemental material spectrum.02491-25-s0001.docx Figures S1 to S9 and Tables S1 to S4.

ASM does not own the copyrights to Supplemental Material that may be linked to, or accessed through, an article. The authors have granted ASM a non-exclusive, world-wide license to publish the Supplemental Material files. Please contact the corresponding author directly for reuse.
