# Supplementary material for: Identification and characterization of two novel Acinetobacter species capable of degrading polyester
Source: Microbiol Spectr. 2026 Apr 9;14(5):e02491-25. doi: 10.1128/spectrum.02491-25 (PMC13141975; doi:10.1128/spectrum.02491-25)
Supplement: Supplemental material — Figures S1 to S9 and Tables S1 to S4. [file spectrum.02491-25-s0001.docx]

Supplementary Information for “Identification and Characterization of Two Novel *Acinetobacter* Species Capable of Degrading Polyester”

Mengli Xia,^1,2,3^ Yuandong Zhao,^1,2,3^ Bo Wu,^1,2,3^ Guoquan Hu,^1,2,3^ Mingxiong He,^1,2,3#^ Yanwei Wang^1,2,3#^

^1^ Biomass Energy Technology Research Centre, Biogas Institute of Ministry of Agriculture and Rural Affairs, Section 4–13, Renmin Rd. South, Chengdu 610041, China

^2^ Graduate School of Chinese Academy of Agricultural Science, No. 12 Zhongguancun South Street, Haidian District, Beijing 100081, China

^3^ Key Laboratory of Development and Application of Rural Renewable Energy, Ministry of Agriculture and Rural Affairs of the People's Republic of China, Section 4–13, Renmin Rd. South, Chengdu 610041, China

**Figure S1.** Growth characteristics of strain CAAS 2-6 and CAAS 2-13 under distinct environmental conditions. (a) Colony morphology on agar plates at different incubation temperatures (4–60°C). (b) Growth curves quantified by OD_600_ measurements under varying pH (4.0–10.0), temperature (15–50°C), and salinity (0–10% NaCl) conditions.


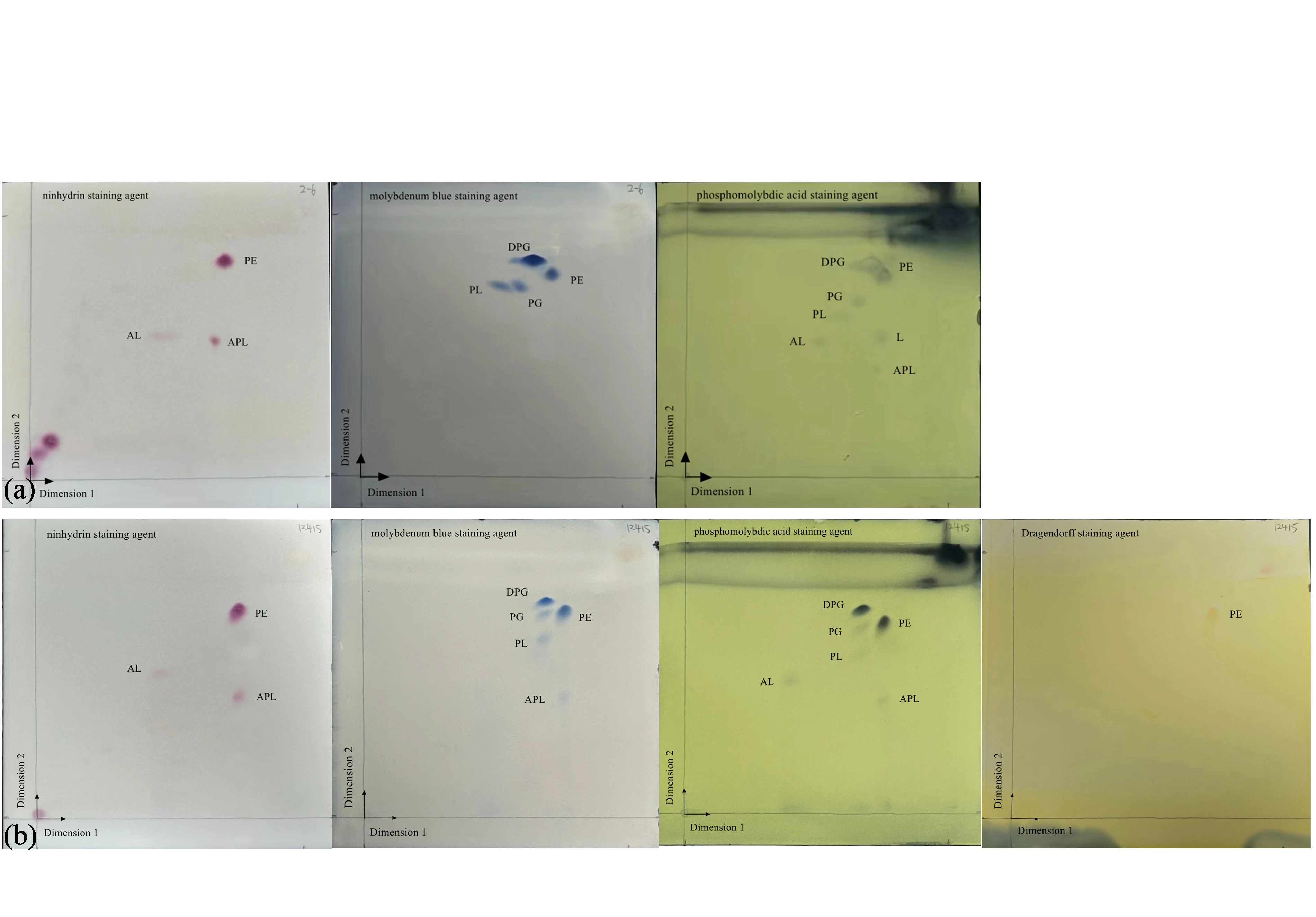


**Figure S2.** Two dimensional TLC plate of polar lipids extracted from strain CAAS 2-6^T^ (a) and *A. gerneri* KCTC 12415^T^ (b) detection with ninhydrinone, molybdenum, anisaldehyde, and molybdatophosphoric acid. The plate was sprayed with 10% (v/v) molybdophosphoric aicd to show all polar lipids present. DPG, diphosphatidylglycerol; PG, phosphatidylglycerol; PE, phosphatidylethanolamine; AL, aminolipid; PL, unidentified phosphoglycolipids; APL, aminophospholipids; L, unknown polar lipids.


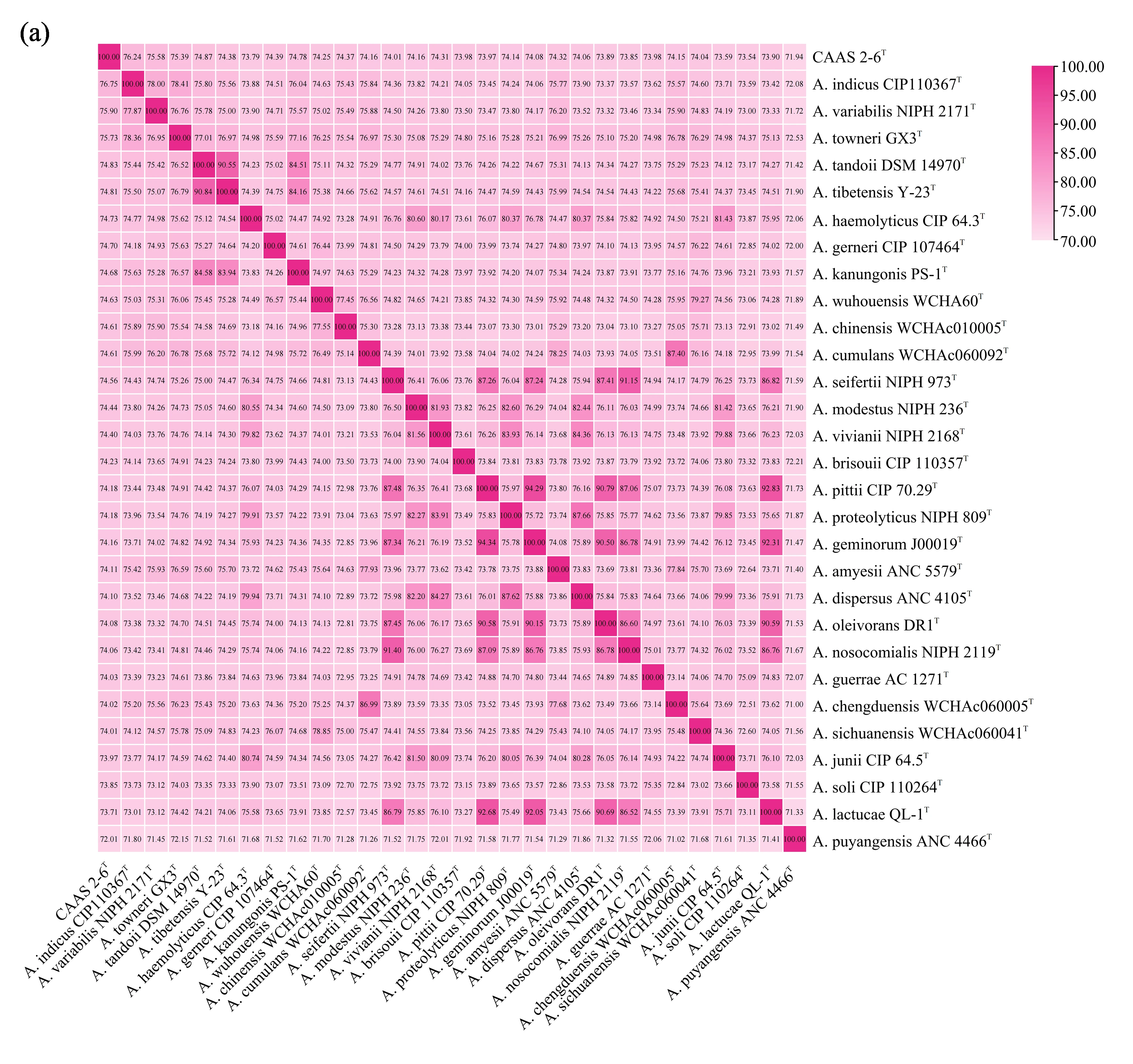


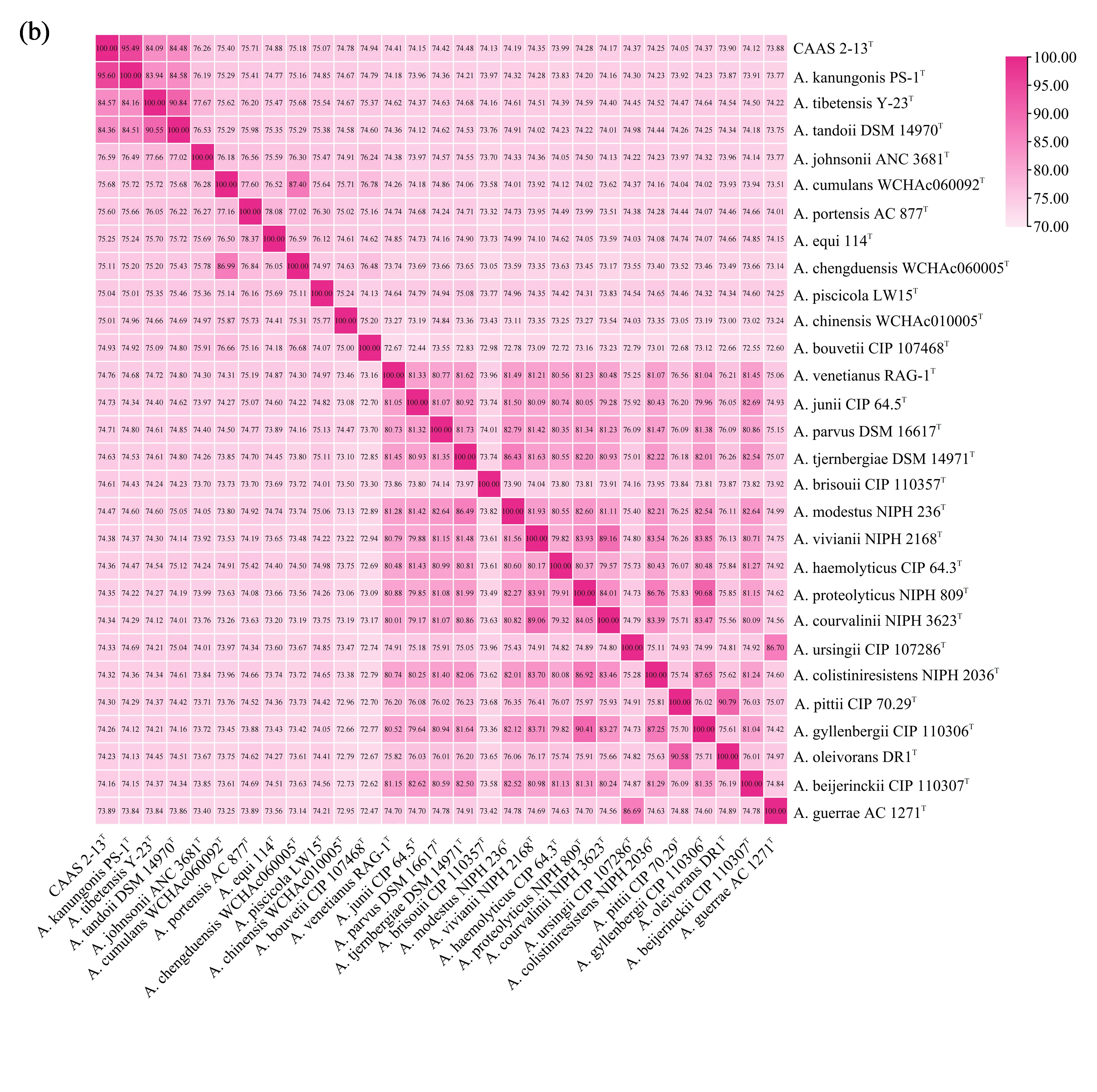

**Figure S3.** Genomic relatedness assessment of CAAS 2-6 and CAAS 2-13. Heatmaps display ANI values with closely related species, calculated by two different algorithms. (a, b) ANIb values for CAAS 2-6 (a) and CAAS 2-13 (b). (c, d) OrthoANI values for CAAS 2-6 (c) and CAAS 2-13 (d), derived from the OAT software.


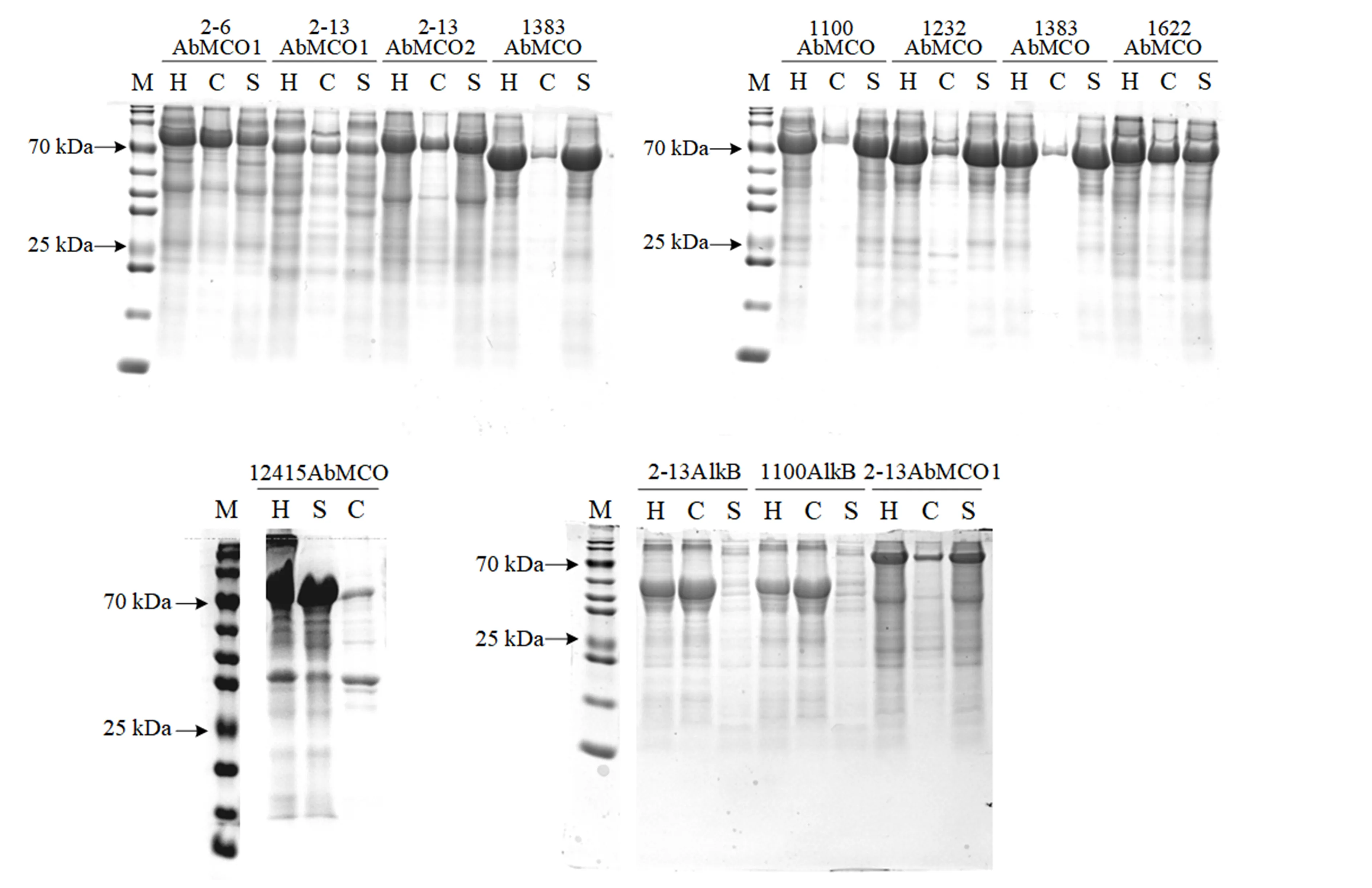


**Figure S4.** SDS–PAGE analysis of recombinant AbMco and AlkB. Heterologously expressed proteins from *Acinetobacter* strains CAAS 2-6^T^, CAAS 2-13^T^ and 5 reference strains (A. gerneri, A. wuhouensis, A. chinensis, *A. sichuanensis*, *A. chengduensis*). M represents molecular size marker, H represents the total cell lysate, S represents the soluble protein, and C represents the insoluble protein. The recombinant AlkB protein has a molecular weight of approximately 46 kDa, while the recombinant AbMco protein ranges from 63 to 70 kDa.


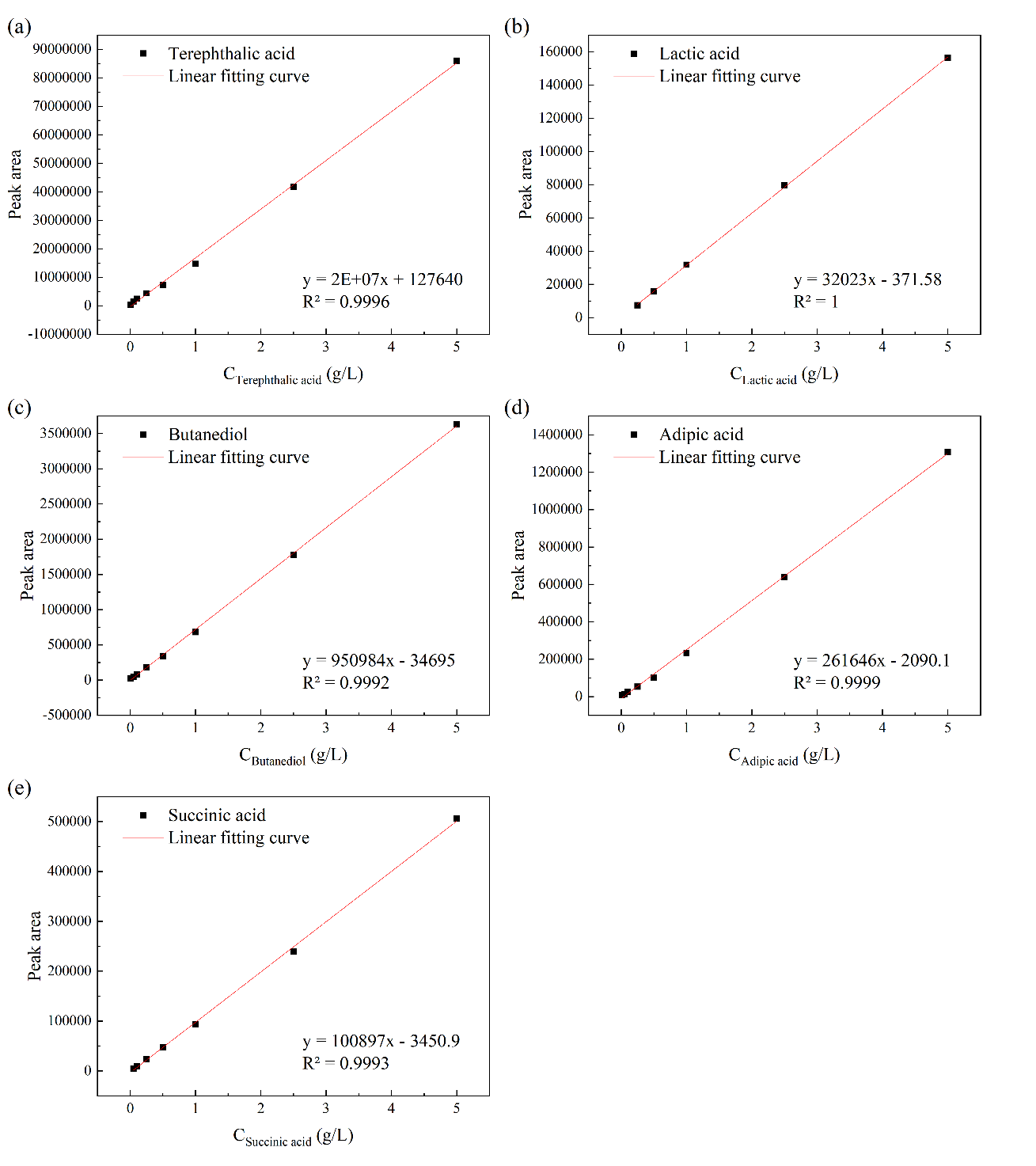


**Figure S5.** Standard curves of terephthalic acid (a), lactic acid (b), butanediol (c), adipic acid (d), and succinic acid (e)


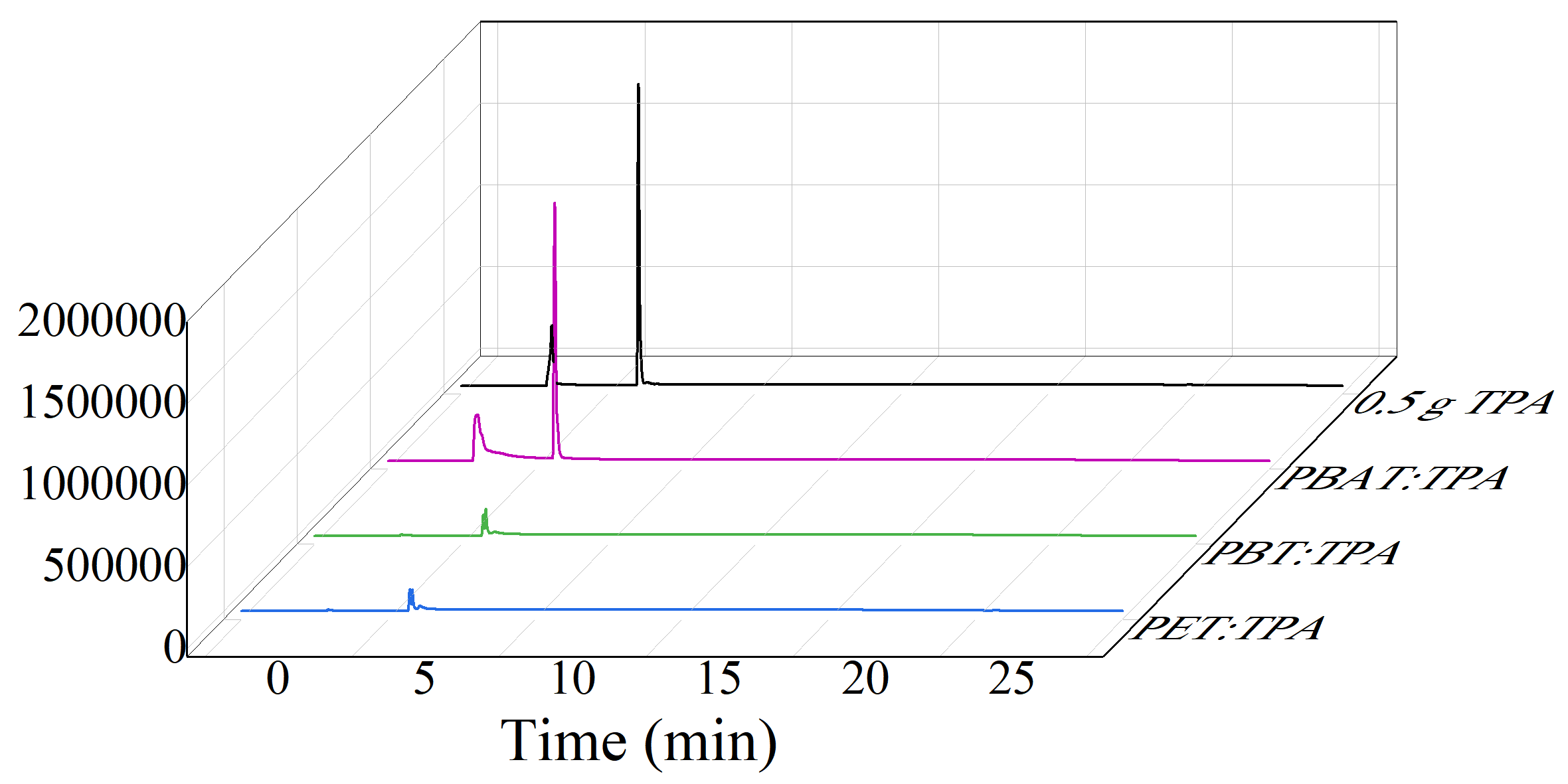


**Figure S6.** Detected chromatographic peaks of TPA generated from the degradation of PBAT/PBT/PET by 2-6AbMCO1 using Shimadzu HPLC. The retention time of TPA was 6.08 min in HPLC analysis.

**Figure S7.** LC-MS analysis of enzymatic degradation products of six plastics by 2-6AbMCO1. (a) and (b) display the total ion current (TIC) chromatograms acquired in positive and negative ion modes, respectively. Owing to the minimal chromatographic peaks detected for PBT and PET, representative mass spectra (m/z vs. relative abundance) at selected scan points are presented solely for PLA (c, g), PBAT (d, h), PBS (e, i), and PBSA (f, j). Key peaks in the mass spectra are annotated with proposed molecular formulas and potential structures, suggesting oligomeric fragments. (k) illustrates the top 20 compounds identified by mass spectral library matching, ranked by relative abundance, with their molecular formulas annotated.


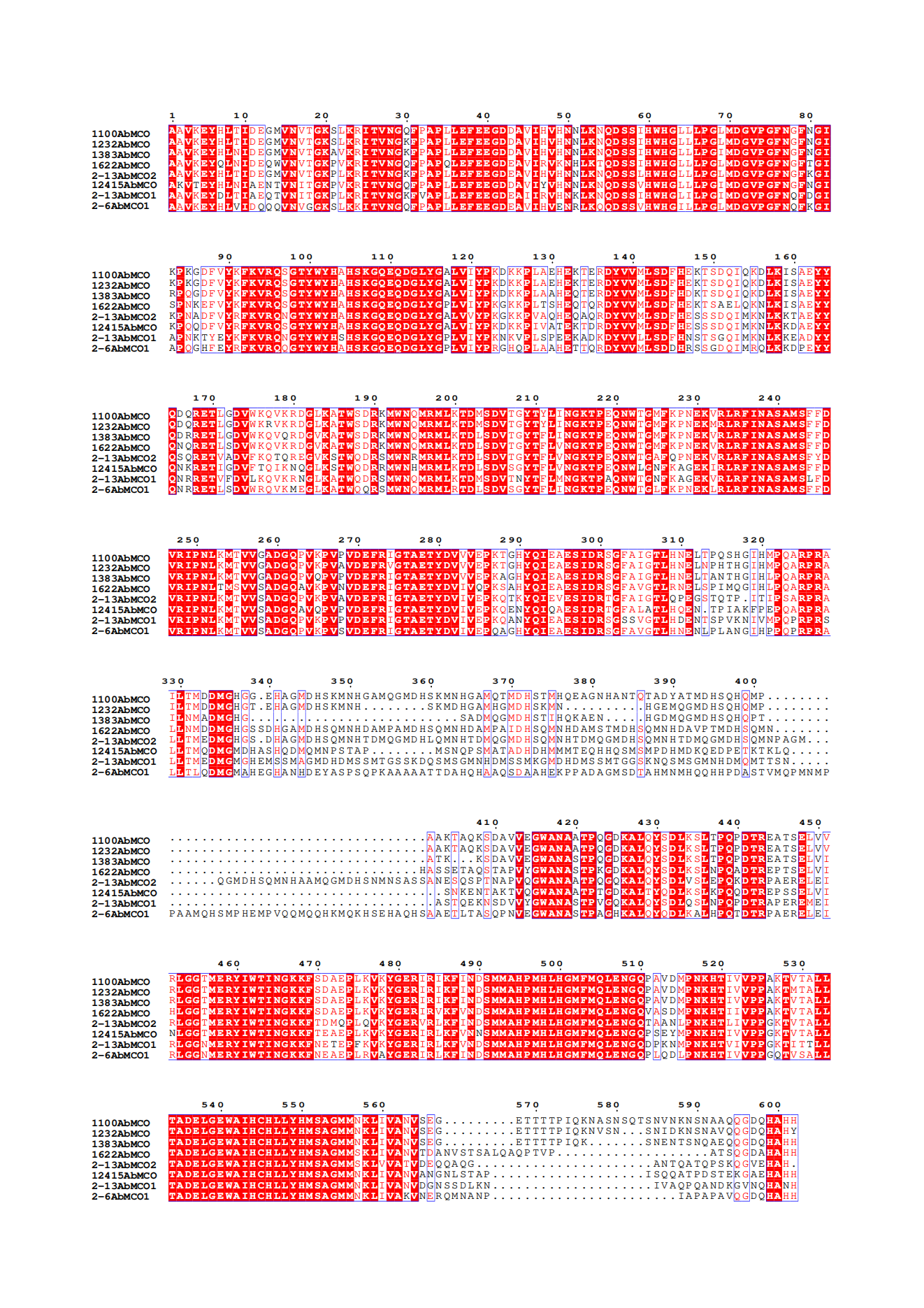


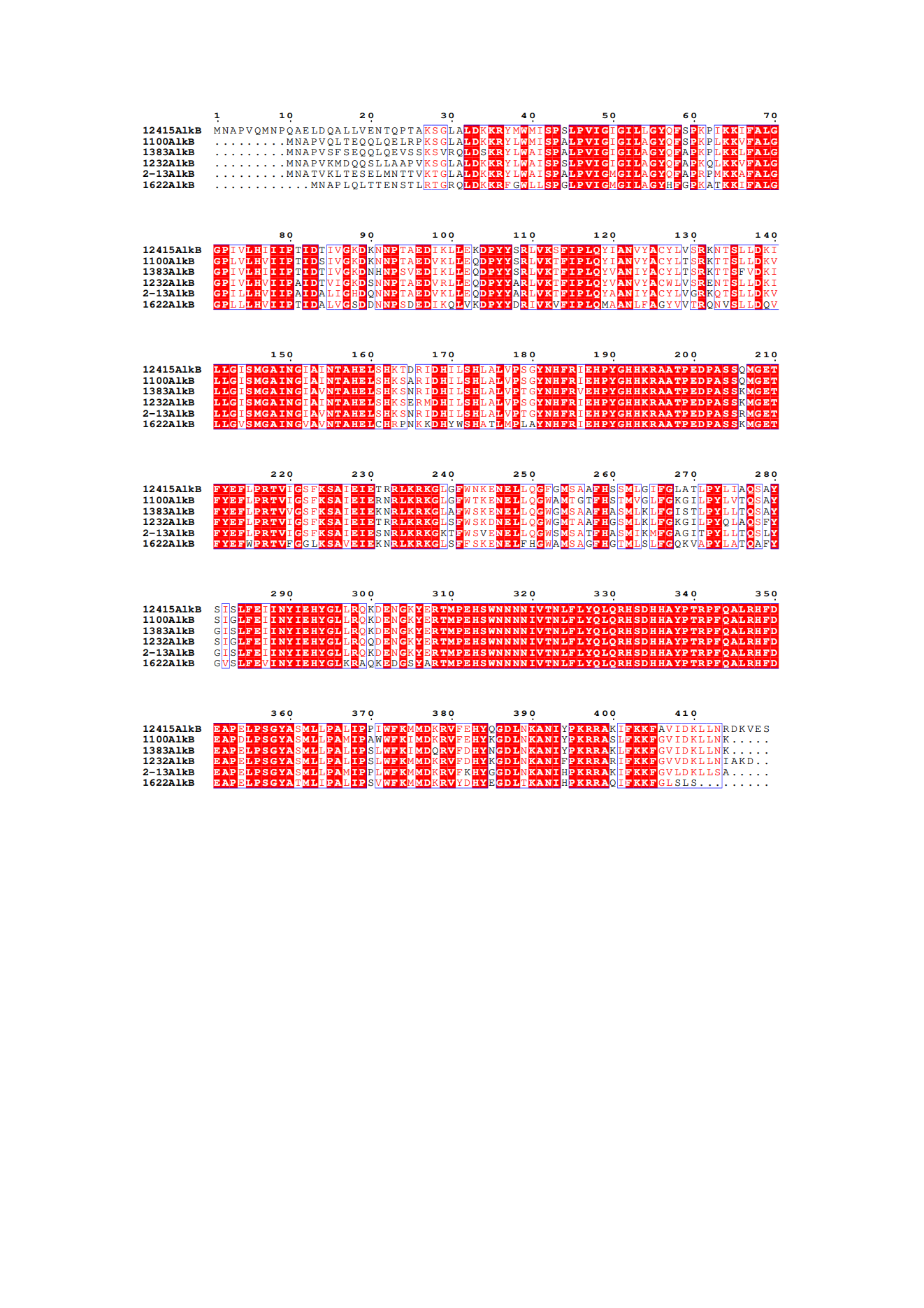


**Figure S8.** Multiple sequence alignment of recombinant AbMCO and AlkB. Color scheme for multiple sequence alignment: Residues in white text on red background indicate strictly conserved positions with 100% identity; red text denotes residues sharing similar physicochemical properties (similarity score > threshold); black text represents non-conserved/variable positions lacking sequence identity or significant similarity.


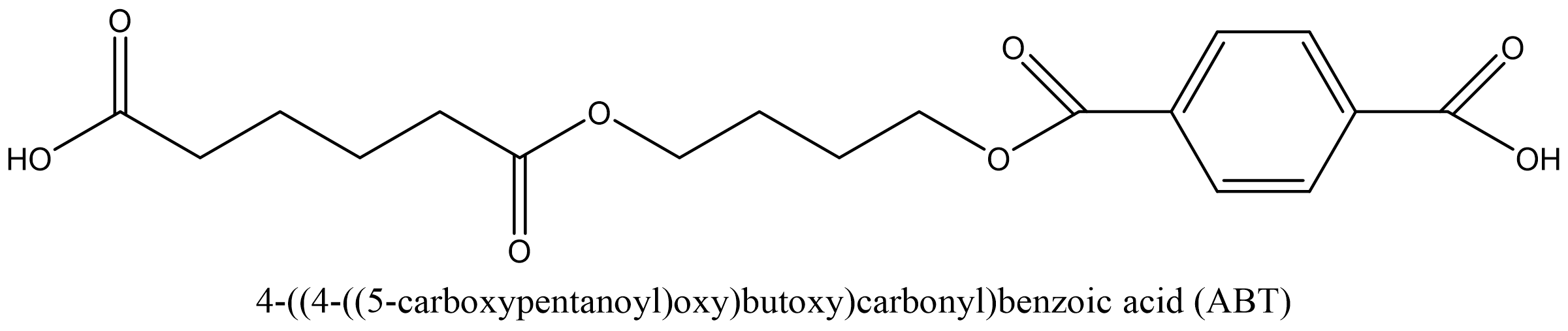


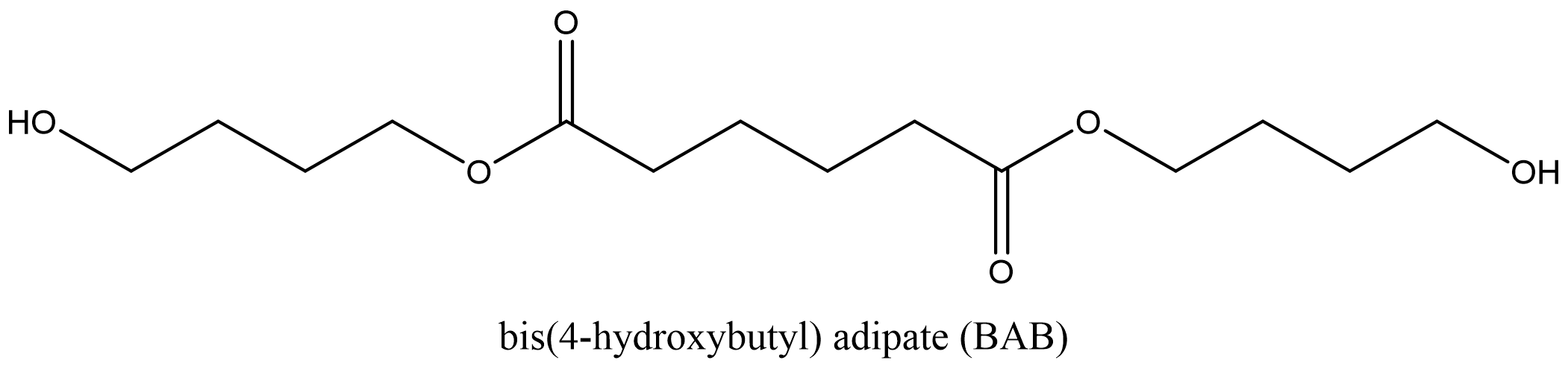


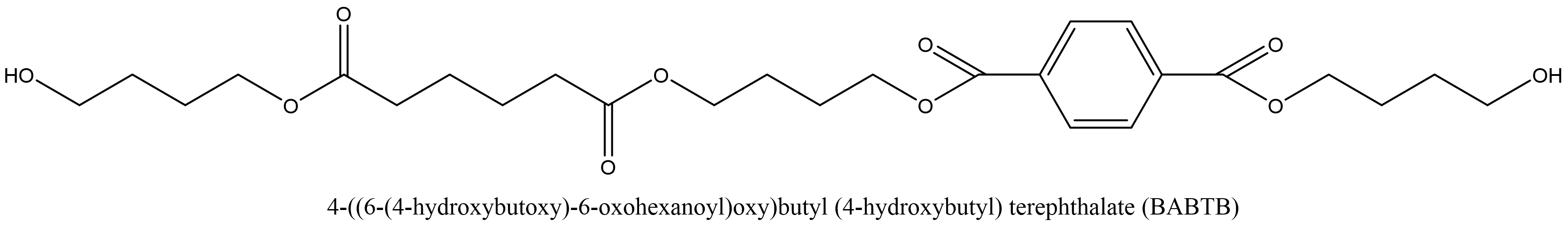


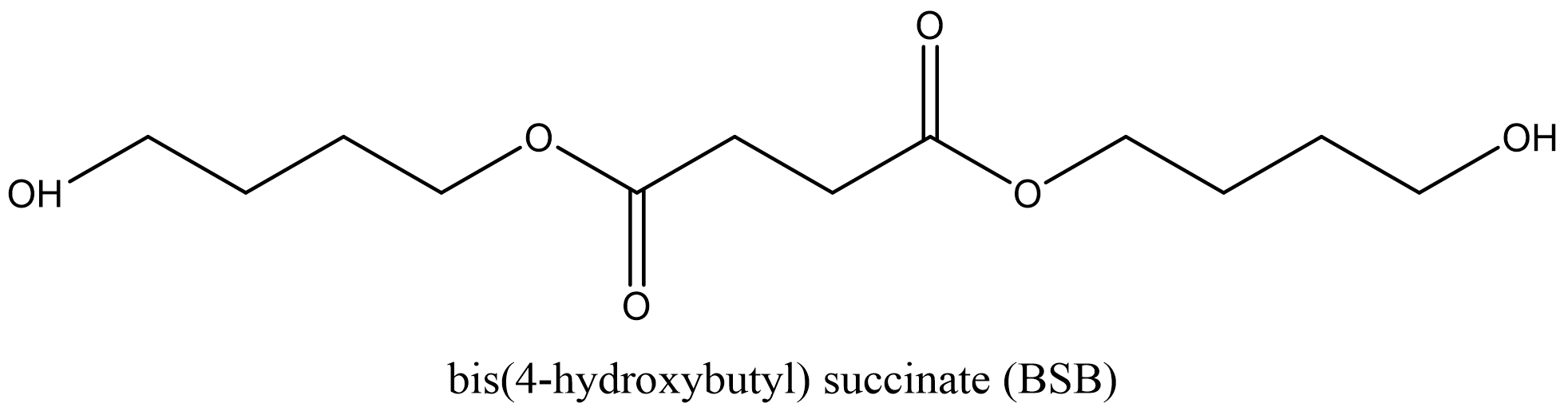


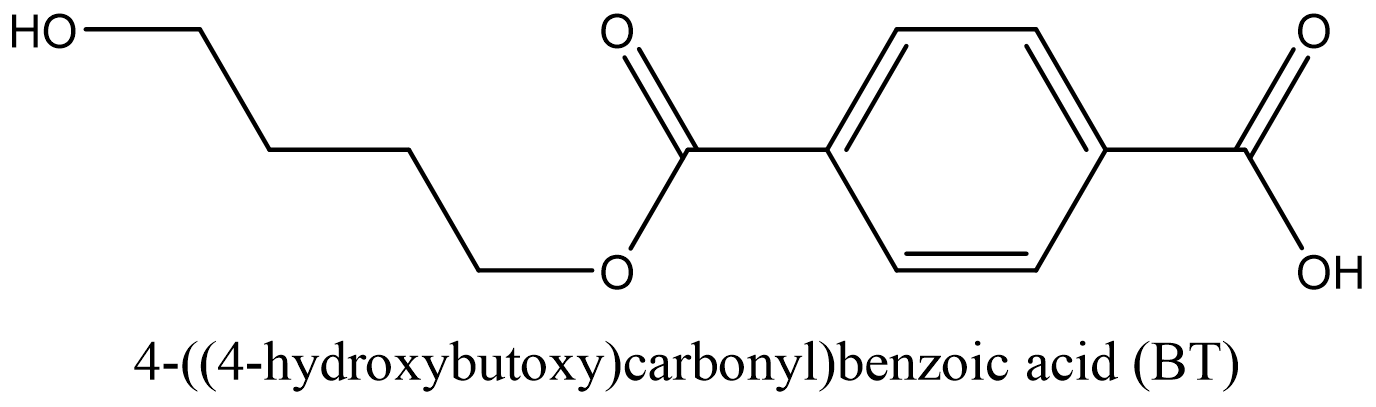


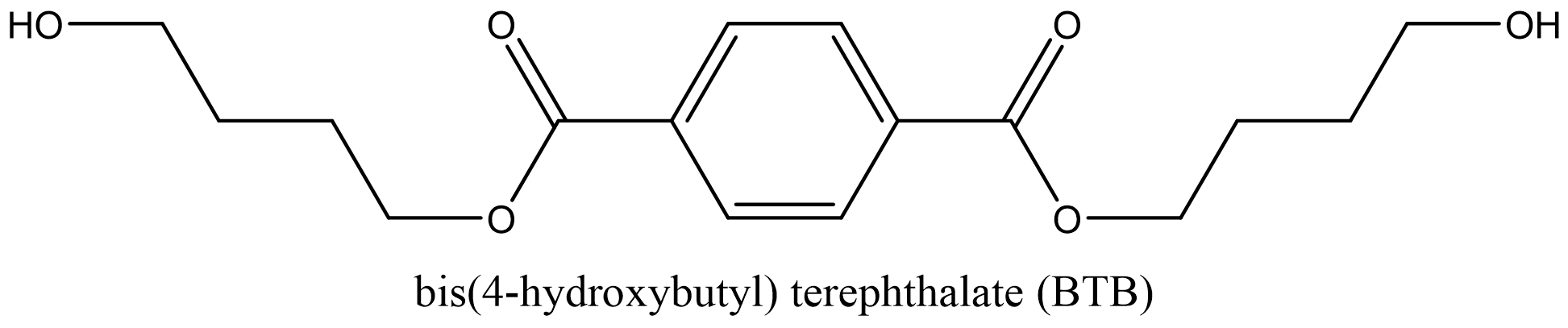


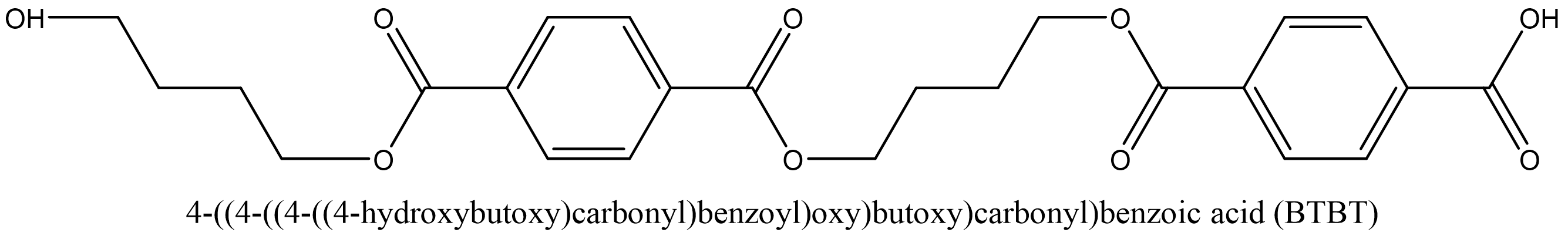


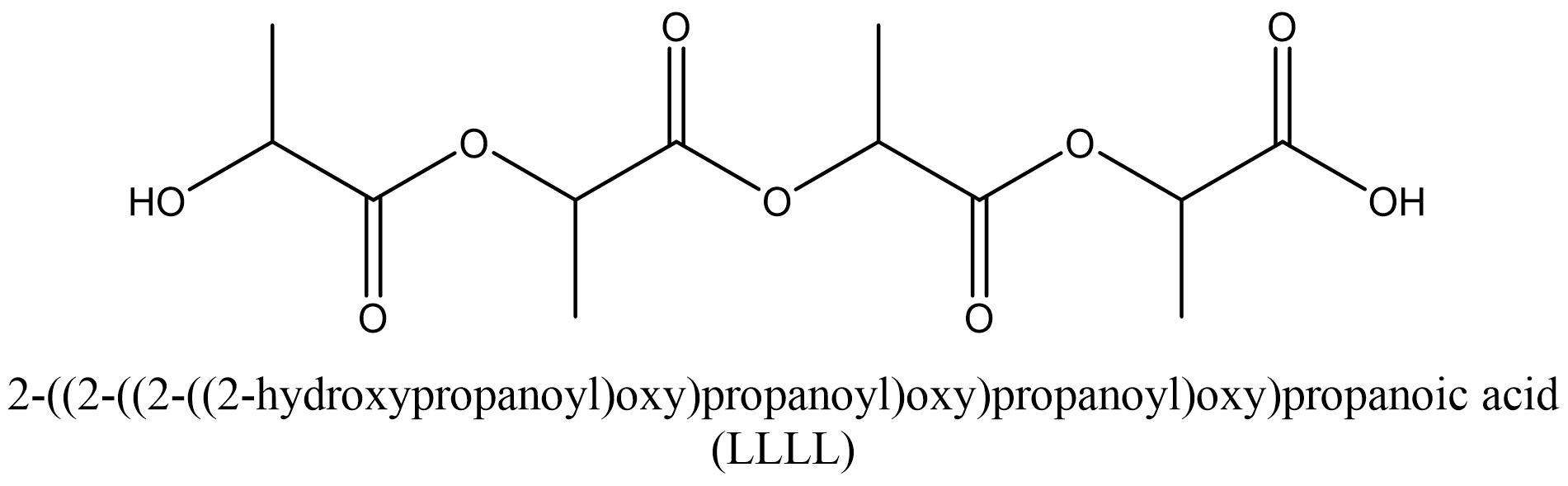


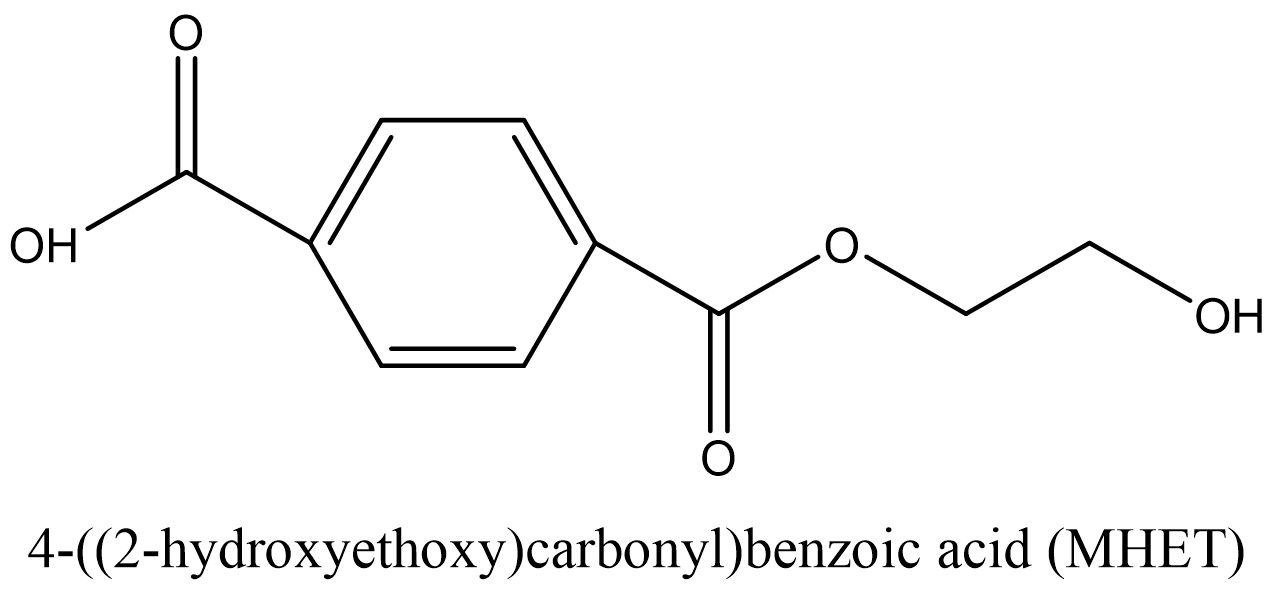


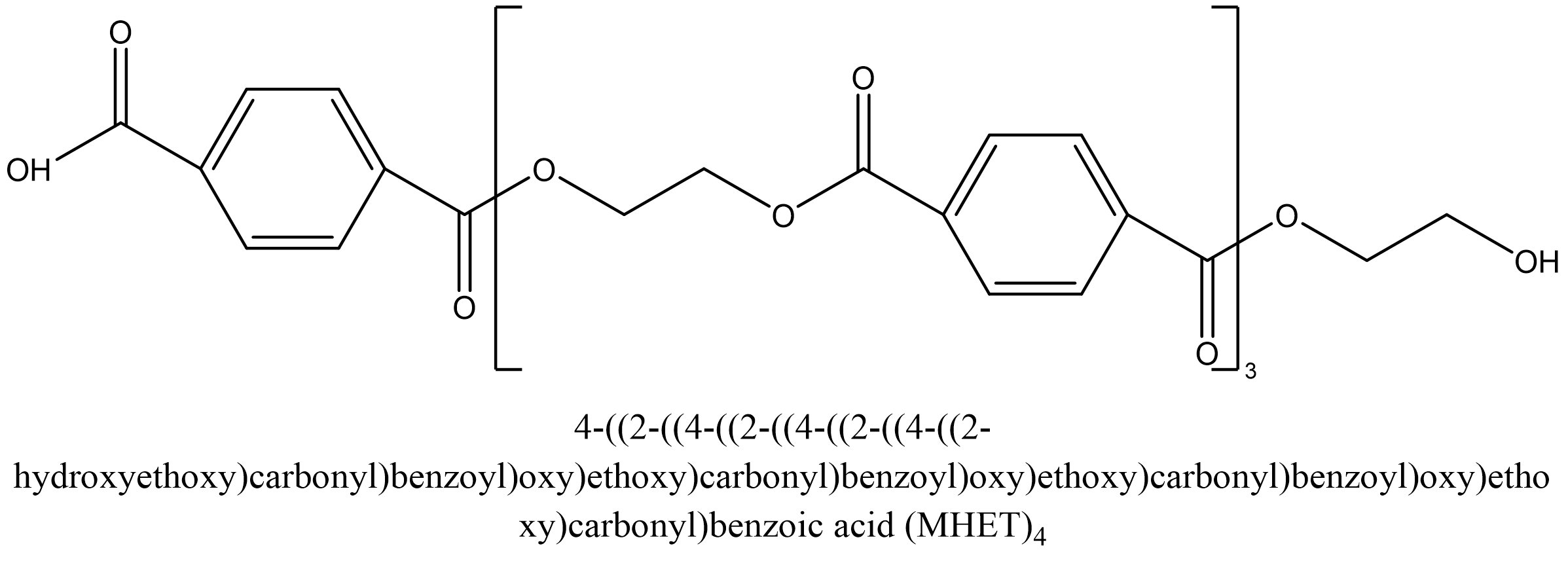


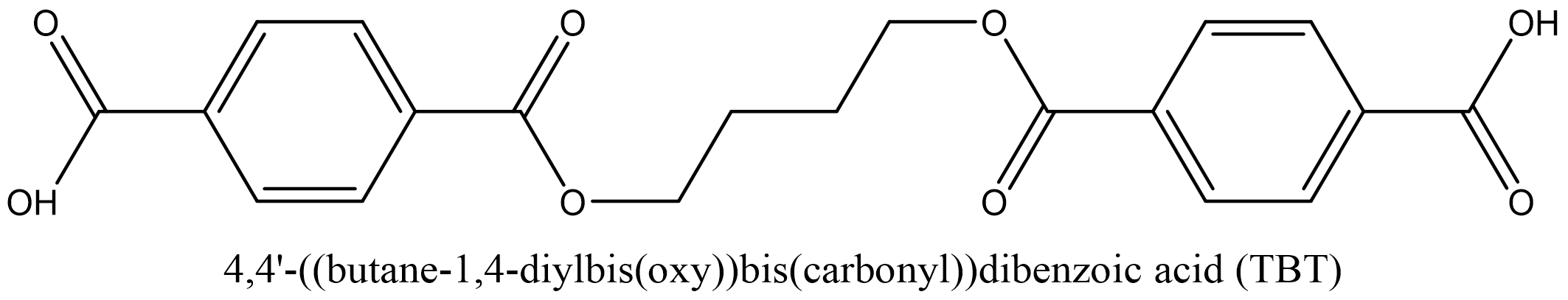

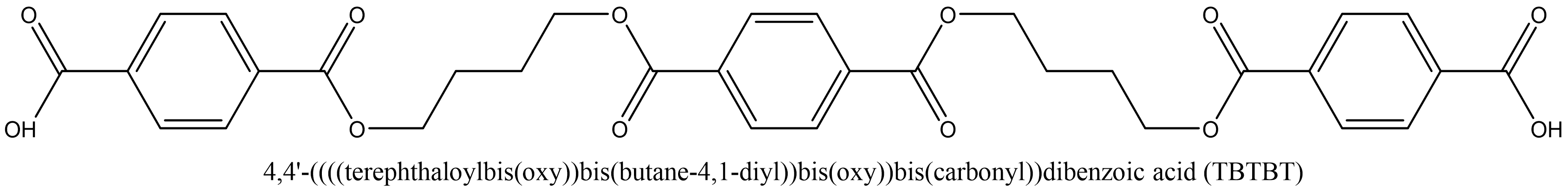


**Figure S9.** Structural formulas of oligomeric substrates used in this study.

**Table S1.** Physiological features of strains CAAS 2-6, CAAS 2-13 and *A. gerneri* KCTC 12415^T^. All dates from this study. +, Positive reaction; –, negative reaction; w, weakly positive reaction.

| **Characteristic** | **CAAS 2-6** | **CAAS 2-13** | **KCTC 12415^T^** |
| --- | --- | --- | --- |
| **API ZYM** |  |  |  |
| Alkaline phosphomonoesterase | W | + | W |
| Lipase | - | W | - |
| Valine arylamidase | W | + | W |
| Cystinol- arylamidase | W | + | + |
| Acid phosphomonoesterase | W | + | W |
| Naphthol-AS-BI-phosphoric acid | W | + | W |
| **API 20E** |  |  |  |
| Sodium citrate | W | W | - |
| L-tryptophane | + | + | W |
| Pyruvate | + | - | - |
| Kohn’s gelatin | - | + | + |
| D-glucose | + | - | + |
| D-mannitol | + | W | - |
| Inositol | + | - | - |
| Sorbitol | + | - | - |
| Rhamnose | W | - | - |
| Sucrose | + | W | - |
| Melibiose | W | - | + |
| Amygdalin | + | - | - |
| L-arabinose | + | - | + |
| **API 20NE** |  |  |  |
| Potassium nitrate | - | - | + |
| Potassium nitrate | - | - | + |
| Gelatin | - | W | W |
| D-glucose | + | - | - |
| Adipic acid | - | - | + |
| Malic acid | - | + | + |
| Citric acid | - | + | W |
| **API 50CH** |  |  |  |
| L-arabinose | - | - | W |
| Ribose | W | - | W |
| D-xylose | - | - | W |
| Galactose | - | - | W |
| Furctose | + | + | W |
| Mannose | + | - | W |
| N-Acetylglucosamine | + | + | - |
| Arbutin | W | - | W |
| Sucrose | + | - | + |
| Starch | + | - | - |

**Table S2.** Genomic assembly results and functional annotation of strains CAAS 2-6 and CAAS 2-13.

| **Property** | **CAAS 2-6** |  | **CAAS 2-13** |  |
| --- | --- | --- | --- | --- |
| Number of contig | 81 | - | 48 | - |
| Number of scaffold | 73 |  | 48 |  |
| Total sequence length | 2,593,745 |  | 3,492,366 |  |
| Coverage (×) | 469 |  | 308 |  |
| GC content% | 44.4 |  | 42.0 |  |
| ORF number | 2,429 | - | 3,332 | - |
| ORF total length | 2,217,186 bp | 85.5 | 3,009,549 bp | 86.2 |
| Longest ORF length | 4,539 bp | - | 5,142 bp | - |
| ORF average length | 912.80 bp | - | 903.23 bp | - |
| Intergenetic region length | 376,559 bp | 14.5 | 482,817 bp | 13.8 |
| 5S rRNA | 1 | 0.0043 | 8 | 0.023 |
| 16S rRNA | 1 | 0.059 | 1 | 0.044 |
| 23S rRNA | 1 | 0.11 | 1 | 0.083 |
| tRNA | 59 | 0.18 | 66 | 0.15 |
| ncRNA | 17 | 0.12 | 18 | 0.088 |
| CRISPRs | 0 | 0 | 0 | 0 |
| NR annotation | 2,344 | 96.5 | 3,273 | 98.2 |
| eggNOG annotation | 2,089 | 86.0 | 2,841 | 85.3 |
| KEGG annotation | 1,437 | 59.2 | 1,773 | 53.2 |
| Swiss-Prot annotation | 1,783 | 73.4 | 2,317 | 69.5 |
| GO annotation | 1,803 | 74.2 | 2,355 | 70.7 |

**Table S3.** Comparative genomic analysis of strain CAAS 2-6 and CAAS 2-13 with the closely species within *Acinetobacter* genus. dDDH results are percentages based on Formula 2: sum of all identities found in high-scoring segment pairs (HSPs) divided by overall HSP length.

| **Features** | **Acc. NO.** | **Assembly type** | **Genome size (bp)** | **G+C mol%** | **Total genes** | **CAAS 2-6** | | **CAAS 2-13** | |
| --- | --- | --- | --- | --- | --- | --- | --- | --- | --- |
|  |  |  |  |  |  | **dDDH (%)** | **ANI (%)** | **dDDH (%)** | **ANI (%)** |
| *Acinetobacter* sp. CAAS 2-6^T^ | JAQIHH000000000 | Scaffold | 3,492,366 | 44.4 | 2,429 | 100 | 100 | 20.3 | 75.7 |
| *Acinetobacter* sp. CAAS 2-13^T^ | JAQIHH000000000 | Scaffold | 3,492,366 | 42.0 | 3,332 | 20.3 | 75.7 | 100 | 100 |
| *A. baylyi* DSM 14961^T^ | GCA_000368685.1 | Scaffold | 3,611,649 | 40.5 | 3,343 | 20.2 | 74.6 | 20.4 | 74.5 |
| *A. beijerinckii* CIP 110307^T^ | GCA_000369005.1 | Scaffold | 3,563,334 | 38.3 | 3,390 | 20.5 | 74.5 | 20.9 | 75.0 |
| *A. bereziniae* LMG 1003^T^ | GCA_000368925.1 | Scaffold | 5,026,532 | 38.3 | 4,709 | 21.4 | 74.9 | 21.5 | 75.5 |
| *A. bouvetii* DSM 14964^T^ | GCA_000368865.1 | Scaffold | 3,388,822 | 45.0 | 3,212 | 20.2 | 74.9 | 20.5 | 75.6 |
| *A. brisouii* CIP 110357^T^ | GCA_000488275.1 | Scaffold | 3,148,512 | 41.7 | 3,026 | 21.3 | 74.9 | 22.0 | 75.0 |
| *A. calcoaceticus* DSM 30006^T^ | GCA_000368965.1 | Scaffold | 3,929,658 | 38.7 | 3,775 | 20.4 | 74.9 | 20.8 | 75.1 |
| *A. chinensis* WCHAc010005^T^ | GCA_002165375.2 | Complete Genome | 3,737,289 | 42.5 | 3,461 | 20.7 | 74.7 | 20.9 | 75.5 |
| *A. colistiniresistens* NIPH 2036^T^ | GCA_000413935.1 | Scaffold | 3,897,684 | 41.3 | 3,651 | 21.3 | 74.9 | 21.0 | 75.3 |
| *A. courvalinii* ANC 3623^T^ | GCA_000369785.1 | Scaffold | 3,992,489 | 42.8 | 3,701 | 20.6 | 75.1 | 20.8 | 75.0 |
| *A. cumulans* WCHAc060092^T^ | GCA_003024525.3 | Complete Genome | 3,710,871 | 40.4 | 3,235 | 21.0 | 75.2 | 21.4 | 76.3 |
| *A. dispersus* ANC 4105^T^ | GCA_000369485.1 | Scaffold | 4,106,934 | 40.5 | 3,890 | 20.7 | 74.9 | 21.0 | 75.1 |
| *A. equi* 114^T^ | GCA_001307195.1 | Complete Genome | 3,054,269 | 34.9 | 2,914 | 21.1 | 74.5 | 21.7 | 76.2 |
| *A. gerneri* DSM 14967^T^ | GCA_000368565.1 | Scaffold | 4,595,172 | 37.9 | 4,284 | 22.3 | 75.1 | 21.6 | 75.2 |
| *A. guerrae* AC 1271^T^ | GCA_009372255.1 | Scaffold | 3,421,735 | 39.2 | 3,293 | 19.7 | 74.7 | 19.9 | 74.7 |

| **Features** | **Acc. NO.** | **Assembly type** | **Genome size (bp)** | **G+C mol%** | **Total genes** | **CAAS 2-6** | | **CAAS 2-13** | |
| --- | --- | --- | --- | --- | --- | --- | --- | --- | --- |
|  |  |  |  |  |  | **dDDH (%)** | **ANI (%)** | **dDDH (%)** | **ANI (%)** |
| *A. guillouiae* CIP 63.46^T^ | GCA_000368145.1 | Scaffold | 4,902,283 | 38.2 | 4,656 | 21.7 | 74.7 | 21.5 | 75.8 |
| *A. gyllenbergii* CIP 110306^T^ | GCA_000414075.1 | Contig | 4,305,849 | 40.9 | 4,188 | 20.5 | 74.9 | 21.0 | 75.2 |
| *A. haemolyticus* CIP 64.3^T^ | GCA_900444835.1 | Contig | 3,598,237 | 39.8 | 3,417 | 21.9 | 74.6 | 21.2 | 74.8 |
| *A. indicus* CIP 110367^T^ | GCA_000488255.1 | Scaffold | 3,211,639 | 45.4 | 3,080 | 22.7 | 77.0 | 21.0 | 76.7 |
| *A. junii* CIP 64.5^T^ | GCA_900444875.1 | Contig | 3,361,057 | 38.8 | 3,194 | 21.0 | 76.1 | 21.5 | 75.3 |
| *A. kanungonis* PS-1^T^ | GCA_009939195.1 | Contig | 3,507,903 | 42.0 | 3,317 | 20.6 | 75.5 | 66.5 | 96.0 |
| *A. lactucae* NRRL B-41902^T^ | GCA_001605885.1 | Scaffold | 3,916,209 | 38.6 | 3,721 | 20.1 | 74.7 | 20.4 | 74.9 |
| *A. modestus* NIPH 236^T^ | GCA_000367965.1 | Scaffold | 3,544,061 | 38.5 | 3,433 | 21.2 | 75.2 | 21.1 | 75.2 |
| *A. nosocomialis* NIPH 2119^T^ | GCA_000368085.1 | Scaffold | 3,906,876 | 38.8 | 3,697 | 20.6 | 74.8 | 20.7 | 74.9 |
| *A. oleivorans* DR1^T^ | GCA_000196795.1 | Complete Genome | 4,152,543 | 38.7 | 3,970 | 20.4 | 74.7 | 20.7 | 75.0 |
| *A. parvus* DSM 16617^T^ | GCA_000368025.1 | Scaffold | 2,912,642 | 41.8 | 2,871 | 21.4 | 75.6 | 21.1 | 75.3 |
| *A. piscicola* LW15^T^ | GCA_002233755.1 | Scaffold | 3,576,146 | 37.2 | 3,319 | 20.6 | 74.8 | 21.1 | 75.9 |
| *A. pittii* CIP 70.29^T^ | GCA_000369045.1 | Scaffold | 3,830,310 | 38.9 | 3,634 | 20.6 | 75.0 | 20.8 | 75.0 |
| *A. portensis* Ac_877^T^ | GCA_009372215.1 | Scaffold | 2,897,281 | 36.6 | 2,813 | 20.1 | 75.2 | 21.2 | 76.6 |
| *A. proteolyticus* NIPH 809^T^ | GCA_000367945.1 | Scaffold | 4,313,783 | 41.3 | 4,008 | 21.2 | 74.8 | 21.1 | 75.0 |
| *A. puyangensis* ANC 4466^T^ | GCA_900096995.1 | Scaffold | 3,781,219 | 40.2 | 3,477 | 21.0 | 76.1 | 20.2 | 72.8 |
| *A. schindleri* CIP 107287^T^ | GCA_000368625.1 | Scaffold | 3,394,558 | 42.3 | 3,257 | 21.7 | 75.8 | 20.8 | 75.6 |
| *A. seifertii* NIPH 973^T^ | GCA_000368065.1 | Scaffold | 4,231,694 | 38.6 | 4,133 | 21.1 | 75.1 | 20.8 | 74.8 |
| *A. sichuanensis* WCHAc060041^T^ | GCA_003024515.2 | Contig | 4,666,610 | 37.0 | 4,609 | 20.5 | 74.8 | 21.4 | 75.7 |

| **Features** | **Acc. NO.** | **Assembly type** | **Genome size (bp)** | **G+C mol%** | **Total genes** | **CAAS 2-6** | | **CAAS 2-13** | |
| --- | --- | --- | --- | --- | --- | --- | --- | --- | --- |
|  |  |  |  |  |  | **dDDH (%)** | **ANI (%)** | **dDDH (%)** | **ANI (%)** |
| *A. soli* CIP 110264^T^ | GCA_000368705.1 | Scaffold | 3,359,986 | 43.2 | 3,161 | 19.8 | 74.5 | 20.2 | 74.1 |
| *A. tandoii* DSM 14970^T^ | GCA_000400735.1 | Scaffold | 4,048,239 | 40.0 | 4,005 | 21.2 | 74.9 | 28.7 | 85.1 |
| *A. tjernbergiae* DSM 14971^T^ | GCA_000374425.1 | Scaffold | 3,692,377 | 38.5 | 3,550 | 20.4 | 74.9 | 21.1 | 75.3 |
| *A. towneri* GX3^T^ | GCA_017498585.1 | Complete Genome | 2,597,534 | 41.5 | 2,718 | 21.4 | 75.9 | 21.6 | 77.3 |
| *A. ursingii* DSM 16037^T^ | GCA_000368825.1 | Scaffold | 3,535,739 | 40.2 | 3,312 | 21.1 | 75.4 | 20.8 | 75.3 |
| *A. variabilis* NIPH 2171^T^ | GCA_000369625.1 | Scaffold | 3,500,761 | 42.0 | 3,365 | 22.2 | 76.6 | 20.7 | 76.0 |
| *A. venetianus* RAG-1^T^ | GCA_000368585.1 | Scaffold | 3,464,338 | 39.2 | 3,342 | 21.6 | 74.5 | 21.6 | 75.2 |
| *A. vivianii* NIPH 2168^T^ | GCA_000369705.1 | Scaffold | 4,084,898 | 41.5 | 3,775 | 21.0 | 75.2 | 21.1 | 75.3 |
| *A. wuhouensis* WCHA60^T^ | GCA_001696605.3 | Complete Genome | 3,960,451 | 38.3 | 3,668 | 21.9 | 74.7 | 21.7 | 76.0 |

**Table S4.** The PCR primers for molecular cloning into the plasmid pET-28a(+). Primer sequences of target enzymes are underlined. Underlined sequences denote overlapping regions.

| **Target** | **Primer** | **Oligonucleotide sequence** |
| --- | --- | --- |
| pET28a(+) backbone | Back-F | AGCTCCGTCGACAAGCTTG |
|  | Back-R | GGATCCGCGACCCATTTGCT |
| *alkB* of strain CAAS 2-13^T^ | 2-13*alkB*-F | AGCAAATGGGTCGCGGATCCATGAATGCAACAGTAAAATTGA |
|  | 2-13*alkB*-R | TGGTGCTCGAGTGCGGCCGCTTATGCGCTAAGCAACTT |
| *alkB* of *A. wuhouensis* WCHA60 = GDMCC 1.1100^T^ | 1100*alkB*-F | AGCAAATGGGTCGCGGATCCATGAATGCTCCAGTCCAA |
|  | 1100*alkB*-R | TGGTGCTCGAGTGCGGCCGCCTACTTATTAAGTAGTTTATCAA |
| *abMCO* of *A. chinensis* WCHAc010005 = GDMCC 1.1232^T^ | 1232*alkB*-F | AGCAAATGGGTCGCGGATCCTCAGTCTTTGGCAATATTTAAA |
|  | 1232*alkB*-R | GGTCGCGGATCCAAGCTTATGAATGCCCCAGTAAAAATGGAT |
| *abMCO* of strain CAAS 2-6^T^ | 2-6*abMCO*-F | AAATGGGTCGCGGATCCATGGCGGTGAAAGAATATCATCTG |
|  | 2-6*abMCO*-R | TGGTGCTCGAGTGCGGCCGCTTAATGATGTGCATGTTGGT |
| *abMCO*1 of strain CAAS 2-13^T^ | 2-13*abMCO*1-F | AAATGGGTCGCGGATCCATGGCTGTTAAAGAATATGATTT |
|  | 2-13*abMCO*1-R | TGGTGCTCGAGTGCGGCCGCTTAATGATTTGCATGTTG |
| *abMCO*2 of strain CAAS 2-13^T^ | 2-13*abMCO*2-F | AAATGGGTCGCGGATCCATGGCGGTCAAAGAATATCATTT |
|  | 2-13*abMCO*2-R | TGGTGCTCGAGTGCGGCCGCTTAGTGCGCATGTTCAAC |
| *abMCO* of *A. wuhouensis* WCHA60 = GDMCC 1.1100^T^ | 1100*abMCO*-F | AAATGGGTCGCGGATCCATGGCTAAAGTCACCGAATATCA |
|  | 1100*abMCO*-R | TGGTGCTCGAGTGCGGCCGCTTAGTGATGTGCATGTTGAT |
| *abMCO* of *A. chinensis* WCHAc010005 = GDMCC 1.1232^T^ | 1232*abMCO*-F | AAATGGGTCGCGGATCCATGGCAGCAGTCAAGGAATAT |
|  | 1232*abMCO*-R | TGGTGCTCGAGTGCGGCCGCTTAGTAATGTGCATGTTGAT |

| **Target** | **Primer** | **Oligonucleotide sequence** |
| --- | --- | --- |
| *abMCO* of *A. sichuanensis* WCHAc060041 = GDMCC 1.1383^T^ | 1383*abMCO*-F | AAATGGGTCGCGGATCCATGGCCGTTAAGGAATATCACCT |
|  | 1383*abMCO*-R | TGGTGCTCGAGTGCGGCCGCTTAGTGGTGTGCATGTTGAT |
| *abMCO* of *A. chengduensis* WCHAc060005 = GDMCC 1.1622^T^ | 1622*abMCO*-F | AAATGGGTCGCGGATCCATGGCGGTCAAAGAATATCAGC |
|  | 1622*abMCO*-R | TGGTGCTCGAGTGCGGCCGCTTAATGGTGCGCATGTG |
| *abMCO* of *A. gerneri* 9A01 = KCTC 12415^T^ | 12415*abMCO*-F | AAATGGGTCGCGGATCCATGGCTAAAGTCACCGAATATCA |
|  | 12415*abMCO*-R | TGGTGCTCGAGTGCGGCCGCTTAGTGATGCGCATGTTCT |
